# Clinical Evidence on the Use of Chinese Herbal Medicine for Acute Infectious Diseases: An Overview of Systematic Reviews

**DOI:** 10.3389/fphar.2022.752978

**Published:** 2022-02-25

**Authors:** Xufei Luo, Yikai Zhang, Huishan Li, Mengjuan Ren, Yunlan Liu, Yunwei Liu, Yilin Zhang, Zhuoran Kuang, Yefeng Cai, Yaolong Chen, Xiaojia Ni

**Affiliations:** ^1^ School of Public Health, Lanzhou University, Lanzhou, China; ^2^ Guangdong Provincial Hospital of Chinese Medicine, Guangdong Provincial, Academy of Chinese Medical Sciences, The Second Clinical School of Guangzhou University of Chinese Medicine, Guangzhou, China; ^3^ The School of Public Health and Management, Guangzhou University of Chinese Medicine, Guangzhou, China; ^4^ Guangdong Provincial Key Laboratory of Research on Emergency in Traditional Chinese Medicine, Guangzhou, China; ^5^ Research Unit of Evidence-Based Evaluation and Guidelines, Chinese Academy of Medical Sciences (2021RU017), School of Basic Medical Sciences, Lanzhou University, Lanzhou, China; ^6^ Institute of Health Data Science, Lanzhou University, Lanzhou, China; ^7^ Evidence-Based Medicine Center, School of Basic Medical Sciences, Lanzhou University, Lanzhou, China; ^8^ WHO Collaborating Centre for Guideline Implementation and Knowledge Translation, Lanzhou, China; ^9^ Guideline International Network Asia, Lanzhou, China; ^10^ Key Laboratory of Evidence Based Medicine and Knowledge Translation of Gansu Province, Lanzhou University, Lanzhou, China; ^11^ Lanzhou University GRADE Center, Lanzhou, China; ^12^ Lanzhou University, An Affiliate of the Cochrane China Network, Lanzhou, China

**Keywords:** Chinese herbal medicine, acute infectious diseases, overview of systematic reviews, COVID-19, public health emergency

## Abstract

**Background:** Acute infectious diseases constitute the most prevalent public health emergency (PHE) in China. Chinese herbal medicine (CHM) has long been used in the treatment of acute infections, but the overall evidence of its benefit and harm has not been comprehensively and systematically evaluated.

**Methods:** We searched CBM, CNKI, Wanfang, PubMed, Cochrane Library, embase and preprint platforms to retrieve systematic reviews (SRs) on CHM for acute infectious. Participants with COVID-19, SARS, H1N1, tuberculosis, bacillary dysentery, mumps, herpangina, hand-foot-and-mouth disease (HFMD), and other acute infectious diseases were included. Interventional group consisting of patients treated with CHM combined with Western medicine or CHM alone. The AMSTAR 2 tool was used to assess the methodological quality of the retrieved studies. Information on interventions, control measures and outcomes of the included studies was extracted, and meta-analyses were qualitatively synthesized.

**Results:** A total of 51 SRs and meta-analyses were eligible for this overview, including 19 for COVID-19, 11 for hand-foot-and-mouth disease, 8 for severe acute respiratory syndrome (SARS), 4 for tuberculosis, 3 for mumps, 2 for bacillary dysentery, 2 for H1N1 influenza and 2 for herpangina. Six systematic reviews were of high quality, all of which were on the use of CHM for COVID-19; 24 were of moderate quality; 10 were of low quality; and 11 were of very low quality. CHM appeared to have potential benefits in improving clinical symptoms and signs for most infections with an acceptable safety profile, and the clinical evidence of the benefits of CHM for acute respiratory infections such as COVID-19, SARS and H1N1 seems more sufficient than that for other acute infections.

**Conclusion:** Overall, CHM, both decoction and Chinese patent medicine, used alone or in combination with conventional medicine may offer potential benefits to relieving symptoms of people with acute respiratory infections. Full reporting of disease typing, staging, and severity, and intervention details is further required for a better evidence translation to the responses for PHE. Future CHM research should focus mainly on the specific aspects of respiratory infections such as its single use for mild infections, and the adjunct administration for sever infections, and individual CHM prescriptions for well-selected outcomes should be prioritized.

## Introduction

Public health emergencies (PHEs) are extraordinary events that are determined to constitute public health risks to other states through the international spread of disease and that potentially require a coordinated international response (World Health Organization, 2005). Acute infectious diseases are among the most common PHEs (World Health Organization, 2017). In China, Chinese herbal medicine (CHM) has a long history of treating acute infections such as smallpox, plague, scarlet fever, cholera, typhoid fever, and malaria (Jiang and Wen, 2021). Given the occurrence and epidemics of infectious diseases across different periods, valuable experience has been accumulated in the use of CHM to fight against infectious diseases, which was often documented in classical literature and monographs (Wang W. et al., 2020). Specifically, *Yellow Emperor’s Internal Classic*, released in approximately 5,000 years ago, was the first publication to find that the occurrence of infectious diseases was closely related to climate change. *Treatise on Cold Attack*, released in the Eastern Han Dynasty, was written after a large-scale epidemic of acute infectious diseases. Doctor Zhongjing Zhang summarized the development of infectious diseases in the book and recorded many classical formulas such as *Xiaochaihu* Decoction and *Maxing Shigan* Decoction, that have been used since then. In late Ming China, with the further deepening of the understanding of infectious diseases in traditional Chinese medicine (TCM), *Systematic Differentiation of Warm Pathogen disease* authored by Doctor Jutong Wu, systematically expounded the general laws of the occurrence, development, evolution and treatment of infectious diseases, in which, *Yinqiao* Powder and *Sangju* Drink, was first documented, and continues to be used for acute upper respiratory disease.

The clinical effectiveness of some classical CHM prescription has been investigated in rigorous randomised controlled trials (RCTs). For example, a single RCT published in *Ann Intern Med* in 2011 suggested that a CHM formula combining *Maxin Shigan* Decoction and *Yinqiao* Power, alone and in combination with an anti-virus pharmacotherapy oseltamivir, can reduce the time for a fever to resolve in patients with H1N1 influenza infection (Wang et al., 2011). Another outstanding example is *artemisia annua L.*, which was recorded in *A Handbook of Prescriptions for Emergencies* (Doctor Hong Ge, Eastern Jin Dynasty) for treating malaria. Later, this CHM formula has been developed to artemisinin, and transferred to clinical practice of malaria, for which Tu Youyou won the Nobel Prize (Tu, 2016).

In modern China, CHM continues to be applied to a wide range of emergent infectious diseases, such as severe acute respiratory syndrome (SARS), H1N1 influenza, and Coronavirus disease 2019 (COVID-19). And there are many clinical trials and systematic reviews of CHM that have been published. However, there has been no comprehensive study describing the status of the treatment of acute infectious diseases with CHM in the manner of critical appraisal. Therefore, we conducted this study to provide an overview of systematic reviews (SRs) of the treatment of infectious diseases with CHM that could serve as a reference for decision-making in this field.

## Methods

We followed the guidance of overviews of reviews published by Hunt et al. (2018). We also reported this overview according to the PRISMA statement (Moher et al., 2009). We have registered this study with the registration DOI: 10.17605/OSF.IO/VZ4S7.

### Inclusion and Exclusion Criteria

#### Study Types Included in This Overview

Systematic reviews (SRs) and meta-analyses, language limited to Chinese and English.

#### Participants

Participants with COVID-19, SARS, H1N1, tuberculosis, bacillary dysentery, mumps, herpangina, hand-foot-and-mouth disease (HFMD), and other acute infectious diseases were included, as identified according to the current list of public PHEs in China (Liu et al., 2019).

#### Interventions

Interventional group consisting of patients treated with CHM combined with Western medicine or CHM alone, where CHM interventions included proprietary Chinese medicine and traditional Chinese medicine decoction. There was no requirement for what should be included in the control group.

#### Outcomes

Outcomes including effectiveness related outcomes which evaluated by the investigator or reported by patients, laboratory tests and radiological imaging, and safety related outcomes such as adverse events, adverse reactions, and toxic scale. The primary outcomes included effectiveness, mortality and adverse events, and secondary outcomes included symptom score, length of stay, laboratory tests and radiological imaging, etc.

#### Exclusion Criteria

Studies were excluded from the search when they were conference abstracts, duplicate publications, unpublished data, and those without full details of a SR.

### Literature Search and Screening

We searched the Chinese Biomedical Literature database (CBM), China National Knowledge Infrastructure (CNKI), Wanfang database, PubMed, Cochrane Library, embase, medRxiv, bioRxiv, China Association of Chinese Medicine, China Association for Acupuncture and Moxibustion, Chinese Medical Journal Network, and Chinese Medicine Journal Network to retrieve relevant systematic reviews/meta-analyses, and the search time was from the date of database creation to 30 October 2020. Before published of this article, we updated the search time to 31 March 2021. For literature screening, two authors read the title and abstract for the initial screening of the literature, and after downloading the full text, it was read and use to further screen the articles, and the results were submitted to a third author for confirmation and verification. The search strategy was specified in Supplementary 1.

### Methodological Quality and Level of Evidence Assessment

The methodological quality of the included studies was evaluated independently by two authors using A MeaSurement Tool to Assess systematic Reviews (AMSTAR 2) (Shea et al., 2017), and a third author assisted in the judgement in cases of disagreement. The methodological quality of AMSTAR2 for systematic review is divided into 16 entries, among which item 2, item 4, item 7, item 9, item 10, item 11, item 13 and item 15 are recommended critical items for determine methodological quality. Considering the specificity of TCM research, we made the following adjustments to the key items. Since some systematic reviews were published before the establishment of the registration platform and the registration platform does not have a Chinese registration language, it was difficult to obtain the protocols of these previous Chinese systematic reviews, so we did not include item 2 as a key entry. Chinese medicine research is mainly published in Chinese language, and most Chinese journal submission systems do not support the presentation of a list of excluded studies, so item 7 was not considered a key entry.

The final evaluation results were classified as 1) “high quality” when there was no or one non-critical weakness, 2) “medium quality” when there was more than one non-critical weakness, 3) “low quality” when there was one critical flaw with or without non-critical weaknesses, or 4) “very low quality” when there was more than one critical flaw with or without non-critical weaknesses.

We also evaluated the level of evidence using the Grading of Recommendations Assessment, Development and Evaluation (GRADE) approach for primary outcomes.

### Data Extraction and Data-Analysis

Two authors independently collected the data on publication information, demographic characteristics, details of the interventions and control measures, outcomes, and statistical results, which were finally checked and confirmed by a third authors. For data analysis, a qualitative integration of the study results was performed for SRs evaluated as having moderate-high quality according to AMSTAR 2.

## Results

### Results of the Searching and Screening

**FIGURE 1:**
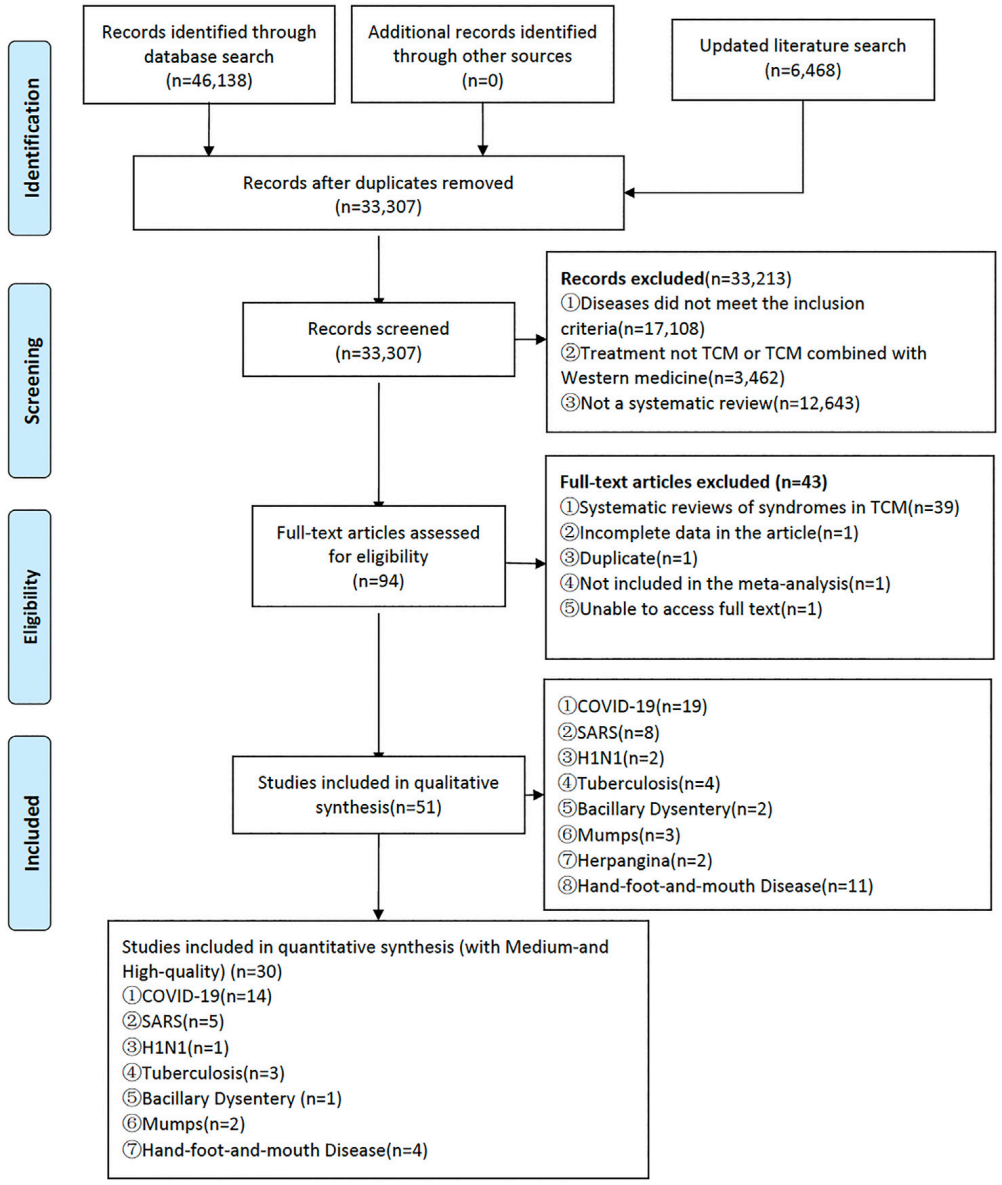
Flow chart of study search and selection.

A total of 46,138 relevant records were obtained from the initial search and 6,468 records were identified from updated search, and after screening, 51 systematic reviews (Liu and Dong, 2021; Liu et al., 2004; Zhang et al., 2004; Zhao et al., 2004; Hao, 2005; Hao et al., 2005; Liu et al., 2005; Chen et al., 2007; Guo et al., 2010; Liu et al., 2012; Ding et al., 2013; Lu et al., 2013; Wang et al., 2013; Xiong et al., 2013; Zhang et al., 2014; Zhang and Wei, 2014; Zhao, 2014; Zhao et al., 2014; Wu et al., 2015; Han, 2016; Li et al., 2016; Liu et al., 2016; Zhang, 2016; Wang et al., 2017; Yan and Gao, 2017; Yue et al., 2017; Jin et al., 2018; Xiong et al., 2019; Ang et al., 2020; Yang et al., 2020a; Yu et al., 2020a; Yang et al., 2020b; Yu et al., 2020b; Fan et al., 2020; Gao et al., 2020; He, 2020; Jin et al., 20201992; Liu et al., 2020; Pang et al., 2020; Qi et al., 2020; Wang et al., 2020; Sun et al., 2020; Wu et al., 2020; Xiong et al., 2020; Yan et al., 2020; Zeng et al., 2020; Zhou et al., 2021a; Zhou et al., 2021b; Luo et al., 2021; Ouyang et al., 2021) were finally included. Among them, 33 (Liu and Dong, 2021; Zhao et al., 2004; Hao, 2005; Hao et al., 2005; Liu et al., 2005; Ding et al., 2013; Lu et al., 2013; Wang et al., 2013; Xiong et al., 2013; Zhang et al., 2014; Zhang and Wei, 2014; Zhao, 2014; Han, 2016; Liu et al., 2016; Zhang, 2016; Wang et al., 2017; Xiong et al., 2019; Yang M. et al., 2020; Yu et al., 2020a; Yang Z. et al., 2020; Yu et al., 2020b; Gao et al., 2020; He, 2020; Qi et al., 2020; Wang S. et al., 2020; Wu et al., 2020; Zhou L. P. et al., 2021; Zhou F. et al., 2021; Ouyang et al., 2021) were written in Chinese, and 18 (Liu et al., 2004; Zhang et al., 2004; Chen et al., 2007; Liu et al., 2012; Zhao et al., 2014; Wu et al., 2015; Li et al., 2016; Ang et al., 2020; Fan et al., 2020; Jin et al., 2020; Liu et al., 2020; Pang et al., 2020; Sun et al., 2020; Xiong et al., 2020; Yan et al., 2020; Zeng et al., 2020; Zhou L. P. et al., 2021; Luo et al., 2021) were written in English. The literature screening process and results are shown in Figure 1. The excluded references are stated in Supplementary 2. The ingredients of the formulas are specified in Supplementary 3.

### Basic Characteristics of the Included Literature

The disease with the largest proportion in the of systematic reviews was COVID-19, with 19 articles (Liu and Dong, 2021; Ang et al., 2020; Yang M. et al., 2020; Fan et al., 2020; Gao et al., 2020; Jin et al., 2020; Liu et al., 2020; Pang et al., 2020; Qi et al., 2020; Wang S. et al., 2020; Sun et al., 2020; Wu et al., 2020; Xiong et al., 2020; Zeng et al., 2020; Zhou L. P. et al., 2021; Zhou F. et al., 2021; Luo et al., 2021; Ouyang et al., 2021), followed by 11 articles on HFMD (Ding et al., 2013; Wang et al., 2013; Xiong et al., 2013; Zhang et al., 2014; Zhang and Wei, 2014; Xiong et al., 2019; Yu et al., 2020a; Yang Z. et al., 2020; Yu et al., 2020b; He, 2020; Yan et al., 2020), 8 for SARS (Liu et al., 2004; Zhang et al., 2004; Zhao et al., 2004; Hao, 2005; Hao et al., 2005; Liu et al., 2005; Chen et al., 2007; Liu et al., 2012), 4 for tuberculosis (Guo et al., 2010; Yan and Gao, 2017; Yue et al., 2017; Jin et al., 2018), 3 for mumps (Zhao, 2014; Wu et al., 2015; Zhang, 2016), 2 for bacterial dysentery (Han, 2016; Wang et al., 2017), 2 for H1N1 (Zhao et al., 2014; Li et al., 2016), and 2 for herpes pharyngitis (Lu et al., 2013; Liu et al., 2016).

The number of RCTs included in each systematic review ranged from 2 to 45. Regarding the type of intervention in the intervention group, TCM combined with Western medicine accounted for the greatest proportion (n = 43, 84.31%) (Liu and Dong, 2021; Fan et al., 2020; Pang et al., 2020; Jin et al., 20201992; Luo et al., 2021; Sun et al., 2020; Zeng et al., 2020; Wang S. et al., 2020; Yang et al., 2020a; Ang et al., 2020; Xiong et al., 2020; Liu et al., 2020; Gao et al., 2020; Qi et al., 2020; Wu et al., 2020; Chen et al., 2007; Liu et al., 2004; Liu et al., 2012; Zhang et al., 2004; Hao, 2005; Hao et al., 2005; Liu et al., 2005; Zhao et al., 2004; Zhao et al., 2014; Li et al., 2016; Jin et al., 2018; Yan and Gao, 2017; Yue et al., 2017; Guo et al., 2010; Wang et al., 2017; Han, 2016; Wu et al., 2015; Zhang, 2016; Zhao, 2014; Lu et al., 2013; Liu et al., 2016; Zhang and Wei, 2014; Zhang et al., 2014; Xiong et al., 2013; Wang et al., 2013; Ding et al., 2013; Yu et al., 2020a; Yang et al., 2020b), with two SRs (3.92%) including studies with CHM alone (Zhao et al., 2014; Yu et al., 2020b) and 6 SRs (11.76%) including studies investigating CHM alone and CHM in combination with Western medicine (Lu et al., 2013; Zhang and Wei, 2014; Zhao, 2014; Liu et al., 2016; Zhang, 2016; Xiong et al., 2019). The most frequently studied herbal preparations were proprietary CHM drugs (*n* = 37, 80.43%), followed by CHM decoction (*n* = 20.43.48%). In terms of pre-defined outcomes, the most used for all diseases were the rate of improvement of clinical symptoms or signs such as fever and cough (*n* = 47, 92.16%), followed by overall effectiveness (*n* = 25, 49.02%), adverse events (*n* = 16, 31.37%), mortality (*n* = 11, 21.57%), and the proportion of lung X-ray shadows absorbed (*n* = 11, 21.57%). Detailed data are shown in Table 1.

**TABLE 1:** Basic characteristics of included literature.

Study	Disease type	Disease stage	Disease classification	Number of included studies	Intervention types	Traditional Chinese medicine treatment	Outcomes	Frequency of the formulas	Adverse event
Fan et al. (2020)	COVID-19	NS	NS	7	Traditional Chinese + H2:H44 medicine + western medicine conventional treatment VS Western medicine treatment	Qingfeitouxiefuzheng decoction	(10) (44) (19)	Qingfeitouxiefuzheng decoction; bid for 10 days; Jinhua Qinggan granules: 15 g tid for 5 days; Toujieqingwen granule: bid for 10 days–15 days; CHM formulae: 200 ml, bid for 7 days; Jiaweidayu granule: tid for 7 days	NS
						Jinhuaqinggan granule			
						Qingfeipaidu decoction			
						Toujieqingwen granule			
						Jiaweidayu granule			
						Shengfutang decoction/Maxinshigan-dayuan decoction			
Pang et al. (2020)	COVID-19	NS	NS	11	Traditional Chinese medicine + western medicine conventional treatment VS Western medicine treatment	Qingfeitouxiefuzheng decoction	(21) (29) (44) (31) (56) (19)	NS	Y
						Jinhuaqinggan granule			
						Toujiequwen granule			
						Qingfeipaidu decoction			
						Maxingxuanfeijiedu Decoction			
						Sufengjiedu capsule			
						Chinese patent medicine + Chinese herbal medicine			
Jin et al. (2020)	COVID-19	NS	NS	5	Traditional Chinese medicine + western medicine conventional treatment VS Western medicine treatment	Qingfeitouxiefuzheng decoction/Lianhuaqingwen granule/Lianhuaqingke granule/Xuebijing injection	(10)	150 ml each time, 2 times a day for 10 days; 6 g each time, 3 times a day for 7 days; 1 bag each time, 3 times a day for 14 days; 50 ml each time, 2 times a day for 7 days	NS
Luo et al. (2021)	COVID-19	NS	NS	RCT:6 CCT:13	Traditional Chinese medicine + western medicine conventional treatment vs Western medicine conventional treatment/Western medicine treatment + Traditional Chinese medicine placebo	Lianhuaqingwen granule	(10) (18) (45) (51) (44) (21) (19)	NS	Y
						Shufengjiedu capsule			
						Touxiequwen granule			
						Reyanning mixture			
						Jinhuaqinggan granule			
						Jiaweidayuan decoction			
						Pneumonia No. 1 formula			
						Modified Qingfeipaidu decoction			
Sun et al. (2020)	COVID-19	NS	NS	7	Traditional Chinese medicine + western medicine conventional treatment vs Western medicine conventional treatment	Shufengjiedu capsule	(10) (19) (29) (18) (25)	TouxieQuwen prescription (2 dose/d); Reyanning mixture (10–20 ml, bid-q6h); Shufengjiedu capsule (2.08 g, tid); Qingfeitouxiefuzheng prescription (1 dose/d); Shufengjiedu capsule (2.08 g, tid); Feiyanyihao prescription or feiyanerhao prescription (1 dose/d); Jinhuaqinggan granule (10 g, tid)	Y
						Touxiequwen granule			
						Reyanning mixture			
						Qingfeixiejiefuzheng formula			
						Feidian No.1 formula/Feidian No.2 formula			
						Jinhuaqinggan granule			
Zeng et al. (2020)	COVID-19	NS	NS	2	Traditional Chinese medicine + western medicine conventional treatment vs Western medicine conventional treatment	Lianhuaqingwen granule	(6) (10) (11) (12) (13) (14) (15) (16) (17) (18) (22)	NS	NS
Wang S. et al. (2020)	COVID-19	NS	NS	7	Traditional Chinese medicine + western medicine conventional treatment VS Western medicine treatment	Lianhuaqingwen granule	(12) (62) (18) (26)	Lianhuaqingwen granule: 6g/bag, 1 bag each time, 3 times a day; 4 tablets/day, tid; 6 g tid	NS
Yang M. et al. (2020)	COVID-19	NS	Ordinary type	RCT:2 NRCT:1	Traditional Chinese medicine + western medicine conventional treatment vs Western medicine conventional treatment	Lianhuaqingwen granule	(3) (25) (19) (36) (48)	NS	N
Ang et al. (2020)	COVID-19	NS	NS	7	Traditional Chinese medicine + western medicine conventional treatment/Traditional Chinese medicine vs Western medicine treatment	Lianhuaqingwen granule	(1) (5) (9) (10) (13) (18) (26) (31) (41) (45)	Lianhua Qingke granules, 1 packet for 3 times daily for 14 days; Shufeng Jiedu capsule, 4 capsules for 3 times daily for 2 weeks; Jinhua Qinggan granules, 2 packets for 3 times daily for 5 days; Toujie Quwen granules, 1 packet per time for 2 times daily for 10–15 days	Y
						Shufengjiedu capsule			
						Touxiequwen granule			
						Jinhuaqinggan granule			
Xiong et al. (2020)	COVID-19	NS	Minor illnesses, major illnesses	18	Traditional Chinese medicine + western medicine conventional treatment vs Western medicine treatment/Western medicine treatment + Traditional Chinese medicine placebo	Maxingshigan decoction/Chailingpingwei decoction/Haoqinqingdan decoction/Huopuxialing decoction/Modified Buzhongyiqi decoction/Pneumonia No. 1 formula/Powerful Pneumonia No. 1 formula/Pneumonia No. 2 formula/Qingfeitouxiefuzheng formula/Shiduyufei formula/Yidubifei formula/Qiwei decoction/Toujiequwen granule/Shufengjiedu capsule/Lianhuaqingwen granule and capsule/Xuanfeizhisou mixture/Shuanghuanglian oral liquid/Yupingfeng granule/Ganluxiaodu decoction/Huoxiangzhengqi liquid/Reyanning mixture/Jinhuaqinggan granule/Xuebijing injection/Tanreqing injection/Shengmai injection/Shenfu injection/Lianhuaqingke granule/Maxingxuanfeijiedu Decoction	(2) (11) (29) (41) (44) (21) (45) (30) (13) (27) (18)	CHM(1dose/d, 10 days); Qingfei Touxie Fuzheng recipe (1dose/d, 10 days); Toujie Quwen granules (1dose/d, 15 days); Jihua Qinggan granules (10 g, tid, 5 days); Reyanning mixture (10–20 ml, bid- q6 h, 7 days); Shufeng Jiedu capsules (2.08g, tid, 10-14 days); Lianhua Qingwen granules (6 g, tid, 7-14 days); Lianhua Qingke granules (1 bag, tid, 14 days); Lianhua Qingwen capsules (1.4 g, tid, 14 days)	Y
Liu et al. (2020)	COVID-19	NS	NS	RCT:4 NRCT:7	Traditional Chinese medicine + western medicine conventional treatment vs Western medicine conventional treatment	Lianhuaqingwen granule	(10) (19) (62) (54) (16) (64)	Diammonium glycyrrhizinate enteric coated capsules (150 mg,tid); Qingfeitouxie fuzhengfang (150 ml,bid); Shufeng Jiedu Capsule (2.08 g,tid); Lianhua Qingwen granules (6 g,tid); Reyanning mixture (10–20 ml,bid); Tongjiequwen granule formula (150 ml,bid); Jinhua Qinggan granules (10 g,tid)	Y
						Shufengjiedu capsule			
						Touxiequwen granule			
						Jinhuaqinggan granule			
						Qingfeitouxiefuzheng decoction			
Gao et al. (2020)	COVID-19	NS	NS	RCT:4 NRCT:8	Traditional Chinese medicine + western medicine conventional treatment vs Western medicine conventional treatment	Lianhuaqingwen granule	(10) (61) (5) (18) (11) (12) (29) (45)	NS	NS
						Shufengjiedu capsule			
						Touxiequwen granule			
						Jinhuaqinggan granule			
						Qingfeixiejiefuzheng decoction			
						Pneumonia 1/pneumonia 2 + conventional treatment			
Liu et al. (2020)	COVID-19	Medical Observation Period	Minor illness, general type	RCT:1 NRCT:6	Traditional Chinese medicine + western medicine conventional treatment vs Western medicine conventional treatment	NS	(12) (25) (26) (29) (41)	NS	NS
Qi et al. (2020)	COVID-19	NS	Ordinary type	RCT:2 NRCT:3	Traditional Chinese medicine + western medicine conventional treatment vs Western medicine conventional treatment	Lianhuaqingwen granule	(10) (36) (5) (6) (11) (12) (36) (44) (19)	Lianhuaqingwen granule: 1 bag per time (6 g), tid	NS
Wu et al. (2020)	COVID-19	NS	Minor/general/severe/critical illnesses	RCT:1 NRCT:7	Traditional Chinese medicine + western medicine conventional treatment vs Western medicine conventional treatment	Lianhuaqingwen granule Shufengjiedu capsule	(12) (13) (18) (26) (27) (29) (25)	NS	NS
Zhou L. P. et al. (2021)	COVID-19	NS	NS	10	Traditional Chinese medicine + western medicine conventional treatment vs Western medicine treatment	Jinhua Qinggan granule	(5) (12) (13) (18) (19)	Jinhua Qinggan granule (3 times a day, once 10 g); Qingfei Touxie Fuzheng recipe (one dose a day, 2 times a day, in the morning and in the evening); Toujie Quwen granules (2 times a day); Lianhua Qingke granule (once 1 bag, 3 times a day); FeiyanYihao Chinese Medicine granules (one dose a day, 2 times a day); Jinyinhua oral liquid (once 60 ml, 3 times a day); Diammonium glycyrrhizinate entericcoated capsule (once 150 mg, 3 times a day); Lianhua Qingwen capsule (once 6 g, 3 times a day); Lianhua Qingwen capsule (4 capsules thrice daily)	Y
						Qingfei Touxie Fuzheng recipe			
						Toujie Quwen granule			
						Lianhua Qingke granule			
						FeiyanYihao Chinese Medicine granule self-made decoction			
						Jinyinhua oral liquid			
						Diammonium glycyrrhizinate enteric-coated capsule			
						Lianhua Qingwen capsule			
Liu et al. (2020)	COVID-19	Medical Observation Period	Minor illness, general type	RCT:1 NRCT:6	Traditional Chinese medicine + western medicine conventional treatment/Traditional Chinese medicine vs Western medicine conventional treatment	Jinhua Qinggan granule	(5) (6) (10) (11) (12) (18) (44)	NS	Y
						Shufeng jiedu granule			
						Jinhua qinggan granule			
						Xuebijing injuction			
Zhou F. et al. (2021)	COVID-19	NS	Minor illness, general type	6	Traditional Chinese medicine + western medicine conventional treatment vs Western medicine treatment	Xuanfei Baidu decoction	(5) (6) (10) (11) (13) (18) (19) (21) (27) (62)	CHM: 1 dose of 300 ml/day, 100ml/time; CHM: 1dose/day, 250 ml/time. bid, 10 days; CHM: 19.4 g, bid; CHM: 200 ml/bag/time, bid	Y
						Maxing Shigan Decoction			
						Keguan⁃1			
						No.1 prescription for pneumonia			
						Hema xingren shigan decoction			
						Qushi Paidu fuzheng decoction			
						Sanreng decoction			
						Xiaochaihu decoction			
Ouyang et al. (2021)	COVID-19	NS	Minor illness, general type	RCT:6 NRCT:4	Western medicine conventional treatment + Traditional Chinese medicine/Western medicine conventional treatment + Placebo + Traditional Chinese medicine vs Western medicine conventional treatment/Western medicine conventional treatment + Placebo	Reyanning mixture	(5) (6) (10) (11) (12) (18) (19) (24) (29) (64)	NA	Y
						Jinhua Qinggan granule			
						Toujie Quwen granule			
						Lianhua Qingwen granule			
						Shufeng Jiedu Capsule			
Chen et al. (2007)	SARS	NS	NS	RCT:15; NRCT:9	Traditional Chinese medicine + western medicine conventional treatment VS Western medicine treatment	NS	(1) (2) (5) (8) (9) (12)	NS	NS
Liu et al. (2004)	SARS	NS	NS	RCT:8; NRCT:8	Traditional Chinese medicine + western medicine conventional treatment vs Western medicine conventional treatment	Feidian No.1/2/3 formula	(2) (3) (4) (5) (9) (11) (14)	TCM: decoction, one dosage daily, for treatment of 21 days; Qiankunning: 6 tablets/time, 4 times daily, for 14 days	NS
						Feidian No.4 formula			
						Guoyao No.2/3 formula			
						Yiqiyang formula/Bufeijianpi formula/Yangyinqingre formula Qianlunning capsule			
						Chuanhuning injection, Shenmai injection, hufeiqingsha decoction/Jieduzhitong capsule/Zhuyinsan capsule			
Liu et al. (2012)	SARS	NS	NS	12	Traditional Chinese medicine + western medicine conventional treatment VS Western medicine treatment	Feidian No.1/2 formula	(2) (3) (5) (6) (7) (8) (9) (13) (14) (15)	National drug No. 2.3 and 4, 2 times/d, 200 ml, for 7–9 days; Kangfeidian No. 1, 2, 3, 2 times/d, 200 ml; potenili 3 times/d, 300 ml	NS
						Feidian No.1 formula			
						Hufeiqingsha decoction			
						Jieduzhitong capsule			
						Zhuyinsanjie capsule			
						Qingshaling spra			
						Feidian No.2/3/4 formula			
Zhang et al. (2004)	SARS	NS	NS	6	Traditional Chinese medicine + western medicine conventional treatment/Traditional Chinese medicine vs Western medicine treatment	Feidian No.1/2/3/4 formula	(2) (6) (7) (8) (9) (16)	NS	NS
Hao et al. (2005)	SARS	NS	NS	RCT:5 CCT:6	Traditional Chinese medicine + western medicine conventional treatment/Traditional Chinese medicine vs Western medicine treatment	Feidian No.1/2/3/4 formula	(12) (27) (63)	NS	NS
						Guoyao No.2/3/4 formula			
						Chuanhupo injection/Shenmai injection/hufeiqingsha decoction Shufengxuanfei formula			
						Xingnaojing injection + Shenmai injection			
						HOUTTUYNIA CORDATA (Chinese pinyin: yuxingcao) injection + Qingkailing injection			
Hao et al. (2005)	SARS	NS	NS	RCT:5 CCT:4	Traditional Chinese medicine + western medicine conventional treatment vs Western medicine conventional treatment	NS	(27)	NS	NS
Liu 2005	SARS	NS	NS	RCT:8 NRCT:8	Traditional Chinese medicine + western medicine conventional treatment/Traditional Chinese medicine vs Western medicine treatment	Yiqiyang formula	(27) (12) (18) (20) (19) (63)	Yiqiyang formula: 1dose/d, 3 weeks; CHM 1 d0se/d, 12 days; Qiankunning 6 tables, 4 times/days, 2 weeks; Guoyao No.2/3/4 formula: 1dose/d; Traditional Chinese medicine SARS No.4 formula: 1 bag, bid; Feidian No.1/2/3/4 formula: 1dose, 2–3 weeks	NS
						Chuanhuning injection/Shenmai injection/Hufeiqingsha decoction/Jieduzhitong capsule			
						Qiankunning capsule			
						Bufeijianpi formula			
						Yangyinqingre formula			
						Guoyao No.2/3/4 formula			
						Feidian No.1/2/3/4 formula			
						Traditional Chinese medicine SARS No.4 formula			
Zhao et al. (2004)	SARS	NS	NS	RCT:5 NRCT:4	Traditional Chinese medicine + western medicine conventional treatment VS Western medicine treatment	Shenmai injection/Hufeiqingsha decoction/Jieduzhitong capsule/Zhuyinsanjie capsule/Qingshaling spray	(27) (4) (12) (18) (5) (23) (19) (63)	NS	NS
						Guoyao No.2/3/4 formula			
						Feidian No.1/2/3/4 formula			
Zhao et al. (2004)	H1N1	NS	NS	5	Traditional Chinese medicine + western medicine conventional treatment VS Western medicine treatment	Lianhuaqingwen granule	(2) (3) (4) (5) (6)	NS	NS
Li et al. (2016)	H1N1	NS	NS	30	Traditional Chinese medicine + western medicine conventional treatment VS Western medicine treatment	Fanggan decoction	(1) (6) (7)	NS	NS
						Lianhuaqingwen capsule			
						Yinqiao decoction			
						Maxingshigan decoction			
						RADIX ISATIDIS(Chinese pinyin:Banlangen) granule			
						Qingkailing injection + Tanreqing injection			
Jin et al. (2018)	Tuberculosis	NS	NS	45	Traditional Chinese medicine + western medicine conventional treatment VS Western medicine treatment	NS	(29) (22) (41) (15) (19)	NS	Y
Yan and Gao (2017)	Tuberculosis	NS	NS	16	Traditional Chinese medicine + western medicine conventional treatment vs Western medicine conventional treatment	Tuberculous pill	(29) (22) (61)	NS	NS
Yue et al. (2017)	Tuberculosis	NS	NS	20	Traditional Chinese medicine + western medicine conventional treatment vs Western medicine conventional treatment	ASTRAGALUS MONGHOLICUS (Chinese pinyin: Huangqi) related Chinese patent medicine, including Feining pill, Jianfeirunpi pill, Yupingfeng Oral liquid, Shuangbai oral liquid, Baidiziyin pill, Buzhongyiqi pill, Zhenqifuzheng granule, Qianggan capsule, Qingjin granule, Bufeihuoxue capsule and Huangqi granule	(39) (18) (15) (61) (19)	NS	Y
Guo et al. (2010)	Tuberculosis	NS	NS	6	Traditional Chinese medicine + western medicine conventional treatment/Traditional Chinese medicine vs Western medicine treatment	Feitai capsule	(29) (39) (22)	NS	NS
						Tuberculin tablet			
						Qibaihe tablet			
						Modified Huangqijianzhong decoction			
						Baozhen decoction			
						Self-made decoction			
Wang et al. (2017)	Bacterial dysentery	Acute phase	NS	12	Traditional Chinese medicine + western medicine conventional treatment vs Western medicine conventional treatment	Modified Baitouweng decoction	(10) (12) (40) (19)	NS	Y
						Zhili decoction			
						Yuli decoction			
						Modified Dachaihu decoction			
						Modified Shaoyao decoction			
						Zhili formula			
						Shaoyao decoction/Baitouweng decoction			
						Dima mixture			
						Gancaozaolian porridge			
						Self-made decoction			
Han (2016)	Bacterial dysentery	Acute phase	Minor, General, Major	28	Traditional Chinese medicine + western medicine conventional treatment vs Western medicine conventional treatment	NS	(10) (50)	NS	NS
Wu et al. (2015)	Mumps	NS	NS	11	Traditional Chinese medicine + western medicine conventional treatment/Traditional Chinese medicine vs Western medicine treatment	ANDROGRAPHIS PANICULATA (Chinese pinyin: chuanxinlian) injection	(4) (12) (10) (9)	Potassium Dehydroandrographolide Succinate Injection: 5–30 mg/(kg.d)	Y
Zhang (2016)	Mumps	NS	NS	7	Traditional Chinese medicine + western medicine conventional treatment VS Western medicine treatment	NS	(10)	NS	NS
Zhao (2014)	Mumps	NS	NS	33	Traditional Chinese medicine + western medicine conventional treatment VS Western medicine treatment	Modified Pujixiaodu decoction + External application of Chinese herbal medicine including RHUBARB(Chinese pinyin: Shengdahuang), TETRADIUM RUTICARPUM(Chinese pinyin: Wuzhuyu), MIRABILITE(Chinese pinyin: Mangxiao) External application of Chinese herbal medicine including CORTEX PHELLODENDRI(Chinese pinyin: Huangbai) and GYPSUM(Chinese pinyin: Shigao) + RADIX ISATIDIS(Chinese pinyin: Banlangen) granule	(10)	NS	Y
						Self-made Fuhuang ointment			
						Reduning injection			
						Shuanghuanglian injection			
						Acupuncture			
						Compound oral mixture of Folium Isatidis (Chinese pinyin: Daqingye) and external application of Cactus			
						Xianfanghuoming decoction + Zijin Cube with vinegar			
						External application of Zhitongxiaoyan ointment + Conventional treatment			
						Shuanghuanglian injection			
						Self-made decoction			
						External application of Wanyin ointment			
						External application of Quzhaling ointment			
Lu et al. (2013)	Mumps	Acute phase	NS	12	Traditional Chinese medicine + western medicine conventional treatment VS Western medicine treatment	Pudilanxiaoyan oral liquid	(10) (19) (44)	NS	Y
Liu et al. (2016)	Herpangina	NS	NS	17	Traditional Chinese medicine + western medicine conventional treatment VS Western medicine treatment	Modified Yinqiao decoction	(10) (19) (12)	NS	NS
						Qingjieliyan decoction			
						Modified Xiexindaochi decoction			
						Self-made Qingjiexiehuang decoction			
						Mixture of Yinqiao decoction			
						Self-made QingQinYinqiao decoction			
						Qingrejieduliyan formula			
						Jieduqinghuo formula			
						Self-made Jieduliyan decoction			
						Self-made Kouchangjing formula			
						Yinqiaohaihe decoction			
						Self-made decoction			
						Self-made Zhitongyanyan decoction			
						Qingyan decoction			
						Niuhuangtianmaliyan powder			
						Qingyanjiedu decoction			
Zhang et al. (2014)	Hand foot mouth disease	NS	Ordinary type	21	Traditional Chinese medicine + western medicine conventional treatment/Traditional Chinese medicine vs Western medicine treatment	Chaihuang granule	(10) (12) (30)	NS	NS
						Modified Gegenqilian decoction			
						Modified Jidaiyu decoction			
						Jieduqingre decoction			
						Jinlan mixture			
						Kangfuxin liquid + Qingrejiedu oral liquid			
						Pudilanxiaoyan oral liquid + Yanhuning injection			
						Qingrexiehuo decoction			
						Sandouyinqiao decoction			
						Yinqiaohuojun decoction			
						Modified Yinqiaomabo decoction			
						Self-made Yinqiaoxiaodu decoction			
						Jinlianqingre granule			
						Self-made Dazi formula			
						Self-made Baidu decoction			
Zhang et al. (2014)	Hand foot mouth disease	NS	Normal type, heavy duty	11	Traditional Chinese medicine + western medicine conventional treatment vs Western medicine conventional treatment	——	(12) (14) (13)	NS	Y
Xiong et al. (2013)	Hand foot mouth disease	NS	NS	6	Traditional Chinese medicine + western medicine conventional treatment vs Western medicine conventional treatment	Xiyanping injection	(10) (14)	NS	Y
Wang et al. (2013)	Hand foot mouth disease	NS	NS	24	Traditional Chinese medicine + western medicine conventional treatment vs Western medicine conventional treatment	Xiyanping injection	(10) (14) (19) (12)	Xiyanping injection: 1–10 mg/kg, iv, qd	Y
Ding et al. (2013)	Hand foot mouth disease	NS	NS	11	Traditional Chinese medicine + western medicine conventional treatment vs Western medicine conventional treatment	Xiyanping injection	(12) (14) (19) (23)	NS	Y
Yu et al. (2020a)	Hand foot mouth disease	NS	NS	17	Traditional Chinese medicine + western medicine conventional treatment vs Western Medicine/Traditional Chinese medicine	Reduning injection/Tanreqing injection/Xiyanping injection/Yanhuning injection	(10) (12) (14) (19) (23)	Reduning injection: 0.3–15 ml/kg, qd; Tanreqing injection: 0.3–0.5 ml/kg, qd; Xiyanping injection: 0.2–10 ml/kg, qd; Yanhuning injection: 5–10 ml/kg, qd	Y
Yang Z. et al. (2020)	Hand foot mouth disease	NS	NS	24	Traditional Chinese medicine + western medicine conventional treatment vs Western medicine conventional treatment	Lanqin oral liquid	(10) (12) (14) (19) (23)	NS	Y
Yan et al. (2020)	Hand foot mouth disease	NS	NS	5	Traditional Chinese medicine + western medicine conventional treatment vs Western medicine conventional treatment	Jinlianqingre effervescent tablets/Jinzhen oral liquid/Kangbingdu oral liquid/Reduning injection/Xiyanping injection	(12) (26) (14) (19)	NS	Y
Xiong et al. (2019)	Hand foot mouth disease	NS	NS	11	Traditional Chinese medicine + western medicine conventional treatment VS Western medicine treatment	Tanreqing injection/Xiyanping injection/Reduning Injection	(10) (12) (26) (14) (19)	Tanreqing injection: 0.3–0.5 ml/kg, 5-10 days; Xiyanping injection: 5–10 mg/kg, 3-10 days; Reduning Injection: 1-5 years, 0.5 ml/kg; 6–10 years, 10 ml; 11–13 years 15ml, 3-10 d	Y
He (2020)	Hand foot mouth disease	NS	NS	14	Traditional Chinese medicine + western medicine conventional treatment VS Western medicine treatment	Xiyanping injection + Chinese patent medicine (Lanqin oral liquid/Kangfuxin liquid/Pudilan oral liquid/Jinhoujian spray/Tanreqing injection)	(10) (12) (14) (19) (37)	NS	NS
Yu et al. (2020b)	Hand foot mouth disease	NS	NS	26	Traditional Chinese medicine vs Western medicine treatment/Traditional Chinese medicine	Lanqin oral liquid	(10) (12) (14) (19) (30) (57)	Fuganlin oral liquid: 10 ml, tid; Huangzhihua oral liquid:10 ml, tid or 5–20 ml, bid; Kangbingdu oral liquid: 10 ml tid; Huangqin oral liquid:10 ml, tid; Pudilan oral liquid:5–10 ml, tid	Y
						PU Di LAN Xiaoyan oral liquid			
						Yellow Gardenia liquid			
						Fuganlin oral liquid			
						Kangbindu oral liquid			
						Huangqing oral liquid			
						Shuanghuanglian oral liquid			
Outcomes:(1) Anxiety relief									
(2) C reaction protein levels									
(3) Chest tightness disappearance rate									
(4) Complications due to hormone use (secondary bleeding, infection, diabetes, hypertension)									
(5) Cough improvement (cough symptom score, cough disappearance time, cough disappearance rate, number of cough disappearance cases, difference in points before and after cough, cough relief rate, cough duration)									
(6) Cough sputum disappearance rate									
(7) D-di-concentration level									
(8) Diarrhea improvement (diarrhoea disappearance rate, diarrhea remission rate)									
(9) Discharge rate									
(10) Efficiency									
(11) Fatigue improvement (weak disappearance time, fatigue disappearance rate, fatigue improvement rate, fatigue improvement case count, fatigue duration, fatigue symptom integration)									
(12) Fever mitigation (number of cases of fever, fever symptom score, fever disappearance rate, fever time, fever control rate)									
(13) Healing rate									
(14) Healing time for rashes or mouth ulcers									
(15) Hollow improvement (shrink rate, close rate)									
(16) IFN-α									
(17) IL-6 level									
(18) Improvement of pulmonary CT (rate of improvement of CT in the lungs, effective rate of improvement in CT in the lungs, absorption rate of pneumonia, improvement rate of imagery of the lungs, lesions absorption)									
(19) Incidence of adverse reactions (liver damage, diarrhea, nausea and vomiting...)									
(20) Incidence of secondary fungal infections following the use of hormones									
(21) Length of stay									
(22) Lesions absorption rate									
(23) Lung immersion absorption (lung immersion absorption time, lung immersion absorption score, number of cases of lung immersion absorption, pulmonary immersion absorption rate)									
(24) Lymphocyte improvement (number of lymphocytes, lymphocyte toxicity, percentage of lymphocytes)									
(25) Major symptoms and inflammatory markers integral									
(26) Mild to severe (severe conversion rate, number of cases of severe illness) (hand, foot and mouth disease)									
(27) Mortality									
(28) Nausea disappearance rate									
(29) Nucleic acid to negative									
(30) Oral ulcers are cured									
(31) Oxygenation index									
(32) Percentage of neutrophils									
(33) Points for dry throat symptoms									
(34) Progress rate of hand, foot and mouth disease									
(35) Quality of life									
(36) Respiratory Difficulty Disappearance Rate									
(37) Resume feeding time									
(38) Secondary infection rate									
(39) Sputum bacteria turn negative									
(40) Stop the time									
(41) TCM Certificate Improvement									
(42) The duration of the sore throat									
(43) The duration of the virus shedding									
(44) The main clinical symptoms are efficient (fever, fatigue and cough)									
(45) The number of cases of severe to mild illness									
(46) The number of CD4 plus (47) The rate of disappearance of shortness of breath									
(48) The rate of loss of appetite									
(49) The rate of muscular aches and pains disappeared									
(50) The time at which the feces was transferred to Yin									
(51) The time of the nucleic acid cathodic turn									
(52) The time when herpes disappeared									
(53) The time when the snot disappeared									
(54) The time when the symptoms disappear									
(55) Time for cheek swelling									
(56) Time of physical pain									
(57) Time of the disappearance of rash and herpes									
(58) Time when nasal congestion disappears									
(59) Total calcitonin levels									
(60) Total medical journey time									
(61) Total remission of clinical symptoms (main symptom disappearance rate, other symptom disappearance rate, total clinical symptom score, difference before and after total clinical symptom score, symptom integral/clinical symptom disappearance time)									
(62) Total remission of clinical symptoms of COVID-19 (main symptom disappearance rate, other symptom disappearance rate, total clinical symptom score, difference before and after total clinical symptom score, symptom score/clinical symptom disappearance time)									
(63)The use of hormone (average hormone dosage, treatment course, average use time, end-of-treatment hormone dosage)									
(64) White blood cell count									

NS: not stated; Y: yes.

Eighteen systematic reviews on COVID-19 that reported on specific drugs showed that the most used proprietary CHM drugs were *Lianhua Qingwen* Granule/Capsule (*n* = 14, 77.78%) and *Shufeng Jiedu* Capsule (*n* = 10, 55.56%), and the most used CHM decoction were *Qingfei Touxie Fuzheng* Decoction (*n* = 7, 38.89%). Six studies that reported specific drugs for SARS showed that the most used prescription was SARS No.2 formula (*n* = 6, 75.00%), SARS No.1 formula (*n* = 5, 62.50%), SARS No.3 formula (*n* = 5, 62.50%) and SARS No.4 formula (*n* = 5, 62.50%). The two H1N1 SRs used *Lianhua Qingwen* Capsule (*n* = 2,100.00%). The three tuberculosis studies that reported specific drugs showed common use of Astragalus Membranaceus (Chinese pinyin: Huangqi) preparations (*n* = 2). One SR for bacillary dysentery reported the use of CHM decoctions such as *Baitouweng* Decoction, *Shaoyao* Decoction, and *Jiawei Dachaihu* Decoction. The two SRs for mumps that reported specific drugs used *Chuanxinlian* injections, externally applied *Fuhuang* ointment, and *Pujixiaodu* Decoction. The two SRs for herpangina reported specific drugs, including *Pudilan Xiaoyan* Oral Solution and *Yinqiao* Decoction. Ten SRs that reported on specific drugs for HFMD most used herbal injections, such as *Xiyanping* Injection (*n* = 7, 70.00%), *Reduning* Injection (*n* = 3, 30.00%) and *Tanreqing* Injection (*n* = 3, 30.00%). Twenty-three SRs reported safety issues, among which one SR concluded that there were no adverse reactions to CHM. Twenty-one SRs reported adverse events, the most common of which were abdominal distension, diarrhoea, nausea, and vomiting, and poor appetite. Detailed data are shown in Table 1.

### Results of AMSTAR2 Quality Assessment

The results of the AMSTAR2 evaluation showed that of the 51 systematic reviews, three (6.52%) were of high quality (Wang S. et al., 2020; Zeng et al., 2020; Luo et al., 2021), 22 (47.83%) were of moderate quality (Zhang et al., 2004; Zhao et al., 2004; Hao, 2005; Hao et al., 2005; Liu et al., 2005; Zhao, 2014; Zhao et al., 2014; Wu et al., 2015; Wang et al., 2017; Yan and Gao, 2017; Yue et al., 2017; Jin et al., 2018; Xiong et al., 2019; Yang M. et al., 2020; Yu et al., 2020a; Yang Z. et al., 2020; Fan et al., 2020; Gao et al., 2020; Jin et al., 2020; Pang et al., 2020; Sun et al., 2020; Xiong et al., 2020), ten (21.74%) were of low quality (Liu et al., 2004; Chen et al., 2007; Guo et al., 2010; Liu et al., 2012; Han, 2016; Li et al., 2016; Ang et al., 2020; Liu et al., 2020; Qi et al., 2020; Wu et al., 2020), and 11 (23.91%) were of very low quality (Liu and Dong, 2021; Zhao et al., 2004; Lu et al., 2013; Liu et al., 2016; Zhang and Wei, 2014; Zhang et al., 2014; Xiong et al., 2013; Wang et al., 2013; Ding et al., 2013; Yan et al., 2020; He, 2020).

**FIGURE 2:**
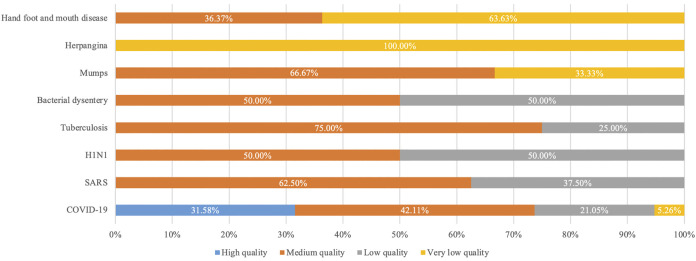
Results of the AMSTAR2 methodological quality evaluation. Abbreviations: AMSTAR2: A MeaSurement Tool to Assess Systematic Reviews 2; COVID-19: Coronavirus disease 2019; HFMD: Hand-foot-and-mouth disease; H1N1: Influenza A subtype H1N1; SARS: Severe Acute Respiratory Syndromes.

Six of the high-quality SRs were on TCMs against COVID-19 (Wang S. et al., 2020; Zeng et al., 2020; Zhou L. P. et al., 2021; Zhou F. et al., 2021; Luo et al., 2021; Ouyang et al., 2021). Most of the medium-quality SRs were on COVID-19 (*n* = 8, 42.11%) (Liu and Dong, 2021; Fan et al., 2020; Pang et al., 2020; Jin et al., 2020; Sun et al., 2020; Yang M. et al., 2020; Xiong et al., 2020; Gao et al., 2020), followed by SARS (*n* = 5, 62.50%) (Zhang et al., 2004; Zhao et al., 2004; Hao, 2005; Hao et al., 2005; Liu et al., 2005), HFMD (*n* = 4, 36.36%) (Xiong et al., 2019; Yu et al., 2020a; Yang Z. et al., 2020; Yu et al., 2020b), tuberculosis (*n* = 3, 75.00%) (Yan and Gao, 2017; Yue et al., 2017; Jin et al., 2018), mumps (*n* = 2, 66.67%) (Zhao, 2014; Wu et al., 2015), H1N1 (*n* = 1, 50.00%) (Zhao et al., 2014) and bacillary dysentery (*n* = 1, 50.00%) (Wang et al., 2017). Among the lower-quality SRs, COVID-19 was also the most frequent disease (*n* = 4, 21.05%) (Ang et al., 2020; Liu et al., 2020; Qi et al., 2020; Wu et al., 2020), followed by SARS (*n* = 3, 37.50%) (Liu et al., 2004; Chen et al., 2007; Liu et al., 2012), H1N1 (*n* = 1, 50.00%) (Li et al., 2016), tuberculosis (*n* = 1, 25.00%) (Guo et al., 2010) and bacillary dysentery (*n* = 1.50.00%) (Han, 2016). The highest number of very low-grade SRs reported on HFMD (*n* = 7, 63.64%) (Ding et al., 2013; Wang et al., 2013; Xiong et al., 2013; Zhang et al., 2014; Zhang and Wei, 2014; He, 2020; Yan et al., 2020), followed by herpangina (*n* = 2, 100.00%) (Lu et al., 2013; Liu et al., 2016), COVID-19 (*n* = 1, 5.26%) (Liu and Dong, 2021), and mumps (*n* = 1, 33.33%) (Zhang, 2016). The summary of AMSTAR 2 assessment is shown in Figure 2. The details of each evaluation item are shown in Supplementary 4.

### Qualitatively Analysis of Medium- And-High-Quality Systematic Reviews

The only two SRs on herpangina was excluded from the data-synthesis due to very low quality. SRs of medium- and high-quality for COVID-19, SARS, H1N1 type influenza, tuberculosis, bacillary dysentery, mumps, and HFMD were included to qualitative data-synthesis. Detailed data are shown in Table 2.

**TABLE 2:** Medium and high-quality literature details.

Study	Diagnosis	Comparison (T vs C)	Outcomes	Estimate (95% CI)	Model	I2	No. participants	No. controlled trials	Level of evidence
Fan 2020	COVID-19	Traditional Chinese medicine + western medicine vs Western medicine	Symptom and inflammatory markers scores	SMD = -1.30 (-2.43, -0.16)	Random	94%	261	3	Low
			C-reactive protein	MD = -11.82 (-17.95, -5.69)	Random	97%	325	5	Low
			Improvement of lung CT	RR = 1.34 (1.19, 1.51)	Random	0%	489	4	Moderate
Pang 2020	COVID-19	Traditional Chinese medicine + western medicine vs Western medicine	Number of severe cases transferred	RR = 0.47 (0.32, 0.69)	Random	0%	989	8	High
			Mortality	RR = 0.50 (0.08, 3.00)	Random	0%	337	2	Moderate
			Length of stay	MD = -7.95 (-14.66, -1.24)	Fixed	——	12	1	Very Low
			Nucleic acid negative conversion rate (%)	RR = 1.08 (0.94, 1.24)	——	——	284	2	Low
			Total score of clinical symptoms	MD = -0.84 (-2.15, 0.47)	Random	92%	250	2	Very Low
			Time of heat removal	MD = -1.20 (-2.03, -0.38)	Random	77%	250	2	Low
			Antipyretic rate (%)	RR = 1.18 (0.88, 1.60)	Random	69%	232	3	Low
			Cough disappearance time	MD = -1.57 (-4.17, 1.03)	Random	94%	250	2	Very Low
			Cough disappearance rate (%)	RR = 1.37 (1.15, 1.64)	Random	0%	264	3	Low
			Weakness disappearance time	MD = -0.33 (-0.78, 0.12)	——	——	200	1	Low
			Weakness disappearance rate (%)	RR = 1.37 (1.02, 1.83)	Random	11%	147	2	Low
			Shortness of breath disappearance rate (%)	RR = 2.20 (1.11, 4.39)	——	——	35	1	Very Low
			Diarrhea remission rate (%)	RR = 0.32 (0.01, 15.49)	Random	87%	30	2	Very Low
			Physical pain disappearance rate (%)	RR = 1.17 (0.73, 1.87)	——	——	30	1	Very Low
			Adverse event incidence rate	RD = 0.03 (-0.02, 0.08)	Random	83%	1,152	8	Moderate
Jin 2020	COVID-19	Qingfeitouxiefuzheng prescription + symptomatic support treatment vs Symptomatic support treatment	Effective rate of pulmonary CT improvement	OR = 2.25 (1.01, 5.01)	——	——	100	——	Very Low
		Lianhuaqingwen granule + symptomatic support treatment vs Symptomatic support treatment		OR = 1.38 (0.91, 2.08)	——	——	397	——	Low
		Lianhuaqingwen granule + symptomatic support treatment vs Symptomatic support treatment		OR = 12.06 (1.37, 106.04)	——	——	57	——	Very Low
		Xuebijing injection + symptomatic support treatment vs Symptomatic support treatment		OR = 9.80 (1.09, 88.23)	——	——	44	——	Very Low
		Lianhuaqingwen granule + symptomatic support treatment vs Qingfei xiefuzheng prescription + symptomatic support treatment		OR = 0.61 (0.25, 1.51)	——	——	249	——	Low
		Lianhuaqingwen granule + symptomatic support treatment vs Qingfei xiefuzheng prescription + symptomatic support treatment		OR = 5.37 (0.53, 54.48)	——	——	83	——	Very Low
		Xuebijing injection + symptomatic support treatment vs Qingfei xiefuzheng prescription + symptomatic support treatment		OR = 4.36 (0.42, 45.27)	——	——	73	——	Very Low
		Lianhuaqingwen granule + symptomatic support treatment vs Lianhuaqingwen granule + symptomatic support treatment		OR = 8.75 (0.96, 79.95)	——	——	230	——	Low
		Xuebijing injection + symptomatic support treatment vs Lianhuaqingwen granule + symptomatic support treatment		OR = 7.11 (0.76, 66.50)	——	——	220	——	Low
		Xuebijing injection + symptomatic support treatment vs Lianhuaqingwen granule + symptomatic support treatment		OR = 0.81 (0.04, 17.89)	——	——	54	——	Very Low
Luo 2020	COVID-19	Traditional Chinese medicine + western medicine vs Western medicine	Cure rate (%)	OR = 2.67 (1.83, 3.89)	Random	0%	792	CCT:7 RCT:3	Moderate
			Improvement of lung CT	OR = 2.43 (1.80, 3.29)	Random	0%	985	CCT:9 RCT:4	Moderate
			Conversion rate of severe cases (%)	OR = 0.40 (0.24, 0.67)	Random	17.1%	840	CCT:8 RCT:3	Moderate
			Nucleic acid negative conversion rate (%)	OR = 2.55 (1.06, 6.17)	Random	56.4%	311	CCT:5	Low
			Cough disappearance rate (%)	OR = 2.95 (1.88, 4.63)	Random	0%	468	CCT:3 RCT:2	Moderate
			Weakness disappearance rate (%)	OR = 2.61 (1.56, 4.34)	Random	0%	368	CCT:3 RCT:1	Moderate
			Fever disappearance rate (%)	OR = 3.17 (1.95, 5.15)	Random	0%	468	CCT:3 RCT:2	Moderate
			Length of stay	MD = -0.46 (-3.87, 2.95)	Random	99.5%	326	CCT:5	Low
			Adverse reactions incidence rate (%)	OR = 1.21 (0.48, 3.07)	Random	43.5%	1,233	CCT:10 RCT:5	Moderate
Sun 2020	COVID-19	Traditional Chinese medicine + western medicine vs Western medicine	Clinical effective rate	RR = 1.21 (1.08, 1.36)	Fixed	0%	273	RCT:2	Low
			Adverse event incidence rate	RR = 1.17 (0.39, 3.52)	Random	62%	681	RCT:7	Low
			Nucleic acid negative conversion rate	RR = 1.49 (1.13, 1.97)	Fixed	0%	185	RCT:3	Low
			Pneumonia Remission rate	RR = 1.27 (1.12, 1.44)	Fixed	0%	415	RCT:4	Low
			White blood cell count	MD = 0.92 (0.07, 1.76)	Random	87%	339	RCT:3	Low
			Lymphocyte count	MD = 0.33 (0.08, 0.57)	Random	76%	188	RCT:3	Low
			Percentage of lymphocytes	MD = 2.90 (2.09, 3.71)	Fixed	0%	273	RCT:2	Low
			C-reactive protein	MD = -12.66 (-24.40, -0.92)	Random	97%	288	RCT:4	Very Low
			IL-6 level	MD = -8.17 (-22.40, 6.06)	Random	73%	166	RCT:2	Very Low
Zeng 2020	COVID-19	Lianhuaqingwen granule + western medicine vs Western medicine	Other symptoms disappearance rate (%)	OR = 6.54 (3.59, 11.90)	Fixed	0%	142	2	Low
			Heating time	OR = -1.04 (-1.60, -0.49)	Random	0%	142	2	Low
			Main symptoms disappearance rate (%)	OR = 3.34 (2.06, 5.44)	Fixed	0%	142	2	Low
			Fever (Main symptoms disappearance rate (%))	OR = 3.64 (1.57, 8.47)	Fixed	0%	142	2	Low
			Cough (Main symptoms disappearance rate (%))	OR = 4.22 (1.73, 10.26)	Fixed	37.9%	142	2	Low
			Weakness (Main symptoms disappearance rate (%))	OR = 2.53 (2.06, 5.44)	Fixed	0%	142	2	Low
			Muscle soreness (Main symptoms/Secondary symptoms disappearance rate (%))	OR = 6.97 (1.47, 33.01)	Random	0%	142	2	Low
			Sputum (Main symptoms/Secondary symptoms disappearance rate (%))	OR = 8.82 (2.48, 31.41)	Random	0%	142	2	Low
			Shortness of breath (Main symptoms/Secondary symptoms disappearance rate (%))	OR = 13.08 (2.60, 65.91)	Random	0%	142	2	Low
			Chest tightness (Main symptoms/Secondary symptoms disappearance rate (%))	OR = 7.17 (1.83, 28.12)	Random	0%	142	2	Low
			Dyspnea (Main symptoms/Secondary symptoms disappearance rate (%))	OR = 2.82 (0.27, 29.18)	Random	0%	142	2	Low
			Nausea (Main symptoms/Secondary symptoms disappearance rate (%))	OR = 1.21 (0.19, 7.81)	Random	0%	142	2	Low
			Loss of appetite (Main symptoms/Secondary symptoms disappearance rate)	OR = 18.07 (0.33, 997.88)	Random	79%	142	2	Low
Wang 2020	COVID-19	Lianhuaqingwen granule + western medicine vs Western medicine	Effective rate of main clinical symptoms	RR = 1.24 (1.12, 1.38)	Fixed	0%	576	5	Moderate
			CT improvement	RR = 1.14 (1.02, 1.28)	Random	53.9%	403	5	Low
			Clinical conversion to severe	RR = 0.48 (0.31, 0.72)	Fixed	10.8%	439	4	Moderate
			Duration of fever	SMD = -0.87 (-1.22, -0.52)	Fixed	0%	186	3	Low
			Clinical symptoms disappearance time	SMD = -0.19 (-1.56, -0.82)	Fixed	0%	151	3	Low
			Length of stay	SMD = -0.61 (-0.91, -0.30)	Fixed	19.6%	416	4	Moderate
Yang 2020	COVID-19	Lianhuaqingwen granule + western medicine vs Western medicine	Fever disappearance rate (%)	RR = 1.76 (1.05, 2.96)	Random	82.8%	197	3	Very Low
			Cough disappearance rate (%)	RR = 1.96 (1.43, 2.68)	Fixed	24.0%	197	3	Low
			Weakness disappearance rate (%)	RR = 1.77 (1.36, 2.30)	Fixed	49.2%	197	3	Low
			Chest tightness disappearance rate (%)	RR = 2.19 (0.89, 5.40)	Fixed	82.8%	197	3	Very Low
			Dyspnea disappearance rate (%)	RR = 4.58 (2.39, 8.79)	Fixed	35.5%	197	3	Low
			Loss of appetite disappearance rate (%)	RR = 1.36 (1.00, 1.84)	Fixed	1.9%	197	3	Low
Xiong 2020	COVID-19	Traditional Chinese medicine + western medicine vs Western medicine/Traditional Chinese medicine placebo + western medicine	Lung CT improved	RR = 1.23 (1.15, 1.32)	Fixed	——	1,402	13	High
			Mortality (%)	RR = 0.34 (0.05, 2.18)	Fixed	0%	463	4	Moderate
			Cure rate (%)	RR = 1.18 (1.13, 1.24)	Fixed	24%	1,523	7	High
			The number of severe to mild cases	RR = 1.34 (0.47, 3.80)	Fixed	0%	167	2	Low
			The number of cases from mild to severe	RR = 0.40 (0.29, 0.56)	Fixed	0%	1,246	11	High
			Length of stay (d)	MD = -1.99 (-3.28, -0.70)	Fixed	——	119	2	Low
			Total score of clinical symptoms	MD = -1.84 (-3.10, -0.58)	Fixed	0%	133	2	Low
			Antipyretic cases	RR = 1.28 (0.98, 1.67)	Random	66%	388	5	Low
			Time of heat removal (d)	MD = -1.36 (-1.8, -0.93)	Random	58%	1,017	10	Low
			Fever symptom score	MD = -0.6 (-0.69, -0.50)	Random	61%	885	3	Low
			Number of cases with cough disappeared	RR = 1.50 (1.26, 1.78)	Fixed	0%	422	6	Low
			Cough symptom score	MD = -0.78 (-1.32, -0.24)	Random	99%	934	4	Low
			Cough disappearance time	MD = -1.42 (-2.82, -0.01)	Random	90%	698	6	Low
			Weakness Number of improved cases	RR = 1.73 (1.39, 2.16)	Fixed	0%	307	5	Moderate
			Weakness Symptom score	MD = -0.70 (-0.98, -0.42)	Random	97%	934	4	Low
			Weakness disappearance time (d)	MD = -1.13 (-2.22, -0.04)	Random	93%	585	4	Low
			Improvement of TCM syndromes (%)	MD = -3.67 (-6.6, -0.73)	Random	86%	225	5	Low
			Nucleic acid negative conversion rate (%)	RR = 1.18 (1.04, 1.34)	Fixed	41%	469	4	Low
			WBC count (109 cell/L)	MD = 0.27 (-0.22, 0.76)	Random	95%	1,151	5	Low
			Lymphotoxicity	MD = 0.24 (-0.04, 0.51)	Random	97%	483	4	Low
			C-reactive protein level (mg/L)	MD = -8.91 (-12.56, -5.27)	Random	97%	1,100	6	Low
			Adverse reactions	RR = 0.93 (0.49, 1.75)	Random	46%	1,069	9	Low
Guo 2020	COVID-19	Traditional Chinese medicine + western medicine vs Western medicine	Total effective rate (%)	RR = 1.31 (1.11, 1.56)	Fixed	0%	138	RCT:2	Very Low
			Difference of total score of clinical symptoms before and after treatment	SMD=0.82 (0.03, 1.61)	Random	84.9%	240	Prospective NRCT:2 RCT:1	Very Low
			Difference of total score of clinical symptoms before and after treatment (RCT subgroup)	SMD=0.20 (-0.17, 0.58)	Random	——	123	RCT:1	Very Low
			Difference of total score of clinical symptoms before and after treatment (RCT subgroup)	SMD=1.17 (0.41, 1.92)	Random	66.6%	117	Prospective NRCT:2	Very Low
			Fever control rate (%)	RR = 1.30 (1.16, 1.45)	Fixed	42.9%	536	Prospective NRCT:3 Retrospective NRCT:1 RCT:2	Low
			Fever integral	SMD=0.76 (-0.57, 2.10)	Random	94.4%	187	Prospective NRCT:1 RCT:2	Very Low
			Fever score (RCT subgroup)	SMD = 1.46 (1.08, 1.83)	Fixed	0%	138	RCT:2	Very Low
			Fever score (NRCT subgroup)	SMD = -0.64 (-1.21, -0.06)	Random	——	49	Prospective NRCT:1	Very Low
			Uration of fever	MD = -1.58 (-1.98, -1.17)	Fixed	9.2%	333	Prospective NRCT:1 Retrospective NRCT:1	Moderate
			Weakness Improvement rate (%)	RR = 1.55 (1.21, 1.99)	Fixed	0%	368	Prospective NRCT:2 Retrospective NRCT:3	Moderate
			Weakness Symptom score	SMD = 1.49 (0.68, 2.30)	Random	83.3%	187	Prospective NRCT:1 RCT:2	Very Low
			Weakness symptom score (RCT subgroup)	SMD = 1.43 (0.14, 2.73)	Random	91.3%	138	RCT:2	Very Low
			Weakness symptom score (NRCT subgroup)	SMD = 1.62 (0.97, 2.27)	Random	——	49	Prospective NRCT:1	Very Low
			Weakness duration	MD=-1.74 (-2.01, -1.48)	Fixed	0%	172	Prospective NRCT:1 Retrospective NRCT:1	Low
			Cough Improvement rate (%)	RR = 1.65 (1.34, 2.04)	Fixed	42.20%	468	Prospective NRCT:2 Retrospective NRCT:1 RCT:2	Low
			Cough Integral difference before and after	SMD=1.95 (1.13, 2.77)	Random	81.40%	187	Prospective NRCT:1 RCT:2	Very Low
			Cough duration	MD=-1.71 (-2.30, -1.12)	Fixed	0%	172	Prospective NRCT:2	Low
			Improvement rate of lung CT	RR = 1.28 (1.04, 1.57)	Random	68.30%	526	Prospective NRCT:2 Retrospective NRCT:3 RCT:2	Low
			Nucleic acid negative conversion rate (%)	RR = 1.43 (0.94, 2.16)	Fixed	0%	138	Prospective NRCT:2	Very Low
			Conversion rate of severe cases (%)	RR = 0.44 (0.26, 0.67)	Fixed	10.30%	842	Prospective NRCT:3 Retrospective NRCT:3 RCT:4	Moderate
Zhou F. et al. (2021)	COVID-19	Traditional Chinese medicine + western medicine conventional treatment vs Western medicine conventional treatment	Adverse reaction	RR = 0.87 (0.67.1.14)	——	——	——	——	Low
			Mortality	RR = 0.33 (0.08.1.34)	——	——	——	——	Low
			Cure rate	RR = 1.15 (CI 1.04.1.26)	Random	60%	976	6	Low
			Lowering body temperature	RR = 1.10 (0.94.1.29)	——	85%	——	9	Low
			Relieving cough	——	——	——	——	9	——
			Improvement in chest CT images	——	——	——	——	5	——
			Deterioration of condition	RR = 0.58 (0.43, 0.77)	——	0%	——	6	Low
			Adverse effects	RR = 0.81 (0.42, 1.57)	——	56%	——	9	Low
Liu 2021	COVID-19	Traditional Chinese medicine + western medicine conventional treatment/Traditional Chinese medicine vs Western medicine conventional treatment	Severe conversion rate	OR = 0.35 (0.18.0.69)	Fixed	0%	326	3	High
			Total effective rate	OR = 2.50 (1.46.4.29)	Fixed	0%	346	3	High
			Pulmonary imaging (CT) improvement rate	OR = 2.27 (1.37.3.77)	Fixed	33%	346	3	Moderate
			Heating duration	SMD = -0.81 (-1.25,-0.38)	Random	75%	414	4	Low
			Fever disappearance rate	OR = 3.05 (1.85.5.01)	Fixed	0%	343	4	Moderate
			Disappearance rate of cough	OR = 2.99 (1.84.4.85)	Fixed	0%	322	4	Moderate
			Disappearance rate of fatigue	OR = 2.60 (1.56.4.33)	Fixed	0%	283	4	Moderate
			Disappearance rate of expectoration	OR = 1.94 (1.19.3.18)	Fixed	56%	315	4	Low
Zhou L. P. et al. (2021)	COVID-19	Traditional Chinese medicine + western medicine conventional treatment vs Western medicine conventional treatment	Healing time of oral ulcer	——	——	——	1,133	7	——
			Adverse reaction	RR = 0.87 (0.67, 1.14)	——	——	812	5	Low
			Cure rate	RR = 1.63 (0.36.7.30)	——	——	——	——	Low
			Total effective rate	RR = 1.25 (0.94.1.67)	——	——	——	——	Low
Ouyang 2021	COVID-19	Western medicine conventional treatment + Traditional Chinese medicine/Western medicine conventional treatment + Placebo + Traditional Chinese medicine vs Western medicine conventional treatment/Western medicine conventional treatment + Placebo	Total effective rate	RR = 1.26 (1.14, 1.40)	Fixed	0%	427	4	Moderate
			Heating duration	WMD = -1.21 (-1.71, -0.71)	Random	55%	414	2	Low
			Disappearance rate of novel coronavirus pneumonia related symptoms	RR = 1.25 (0.88, 1.80)	Random	——	——	5	Moderate
			Pneumonia absorption rate	RR = 1.15 (0.93, 1.43)	Random	84%	——	6	Low
			Disapperance rate of weakness	RR = 1.36 (0.71, 2.62)	Random	75%	——	——	Low
			Disapperance rate of cough	RR = 1.87 (0.58, 6.08)	Random	97%	——	——	Low
			Virus nucleic acid negative rate	RR = 1.47 (1.05, 2.05)	Fixed	0%	——	3	High
			Leukocyte count	RR = 0.74 (0.26, 1.22)	Random	75%	——	2	Low
			Lymphocyte count	RR = 0.21 (0.15, 0.27)	Fixed	0%	——	2	High
			Percentage of lymphocytes	RR = 2.69 (1.92, 3.47)	Fixed	31%	——	2	High
Zhang 2004	SARS	Combination of Chinese and Western medicine vs Western medicine	Mortality (%)	RR = 0.86 (0.22, 3.29)	Random	——	139	6	Low
		GuoYaoNO.2.3.4 formula + westernmedicine vsWestern medicine	Mortality (%)	RR = 0.41 (0.04, 4.78)	Fixed	——	53	6	Very Low
		GuoYaoNO.2.3.4 formula + western medicine vs Western medicine	Secondary infection rate	RR = 0.42 (0.11, 1.62)	Fixed	——	53	6	Very Low
		GuoYaoNO.2.3.4 formula + western medicine vs Western medicine	Lung infiltration and absorption (%)	RR = 5.45 (1.54, 19.26)	Fixed	——	53	6	Very Low
		FeidianNO.1.2.3 formula + western medicine vs Western medicine	Lung infiltration and absorption (%)	RR = 6.68 (2.93, 15.24)	Random	——	139	6	Low
		FufangNo.1 formula + western medicine vs Western medicine	Lung infiltration and absorption (%)	MD = 0.24 (0.02, 0.46)	Fixed	——	40	6	Very Low
		Combination of Chinese and western medicine vs Western medicine	Lung infiltration and absorption (%)	RR = 8.06 (0.4, 163.21)	Fixed	——	59	5	Very Low
		FeidianNo2.3.4 formula + western medicine vs Western medicine	Dyspnea disappearance	RR = 1.50 (0.41, 5.43)	Fixed	——	38	1	Very Low
		FeidianNo.4 formula + western medicine vs Western medicine	Cough disappearance	RR = 1.29 (0.30, 5.43)	Fixed	——	30	1	Very Low
		Combination of Chinese and western medicine vs Western medicine	Average total dosage of hormone (mg)	MD = -39.65 (-116.84, 37.54)	Fixed	——	98	2	Very Low
Hao 2005	SARS	Traditional Chinese medicine + western medicine vs Western medicine	Mortality (%)	RR = 0.24 (0.13, 0.42)	Random	0%	697	9	High
			Average dosage of hormone (mg)	SMD = -1.40 (-2.58, -0.23)	Fixed	95.30%	175	5	Very Low
			Mean heating time	RD = -0.65 (-1.45, -0.15)	Random	21.10%	73	4	Very Low
Hao, Hong 2005	SARS	Traditional Chinese medicine + western medicine vs Western medicine	Mortality (%)	RR = 0.24 (0.13, 0.43)	Random	——	599	9	High
Liu 2005	SARS	Traditional Chinese medicine + western medicine vs Western medicine/p lacebo	Mortality (%)	RCT:RR = 0.32 (0.12, 0.91) NRCT:RR = 0.27 (0.12, 0.61)	Random	——	RCT:294 NRCT:486	RCT:5 NRCT:6	High
			Time of heat removal(d)	MD = -0.83 (-1.3, -0.35)	Fixed	——	182	3	Low
			Symptom relief time (d)	MD = -1.23 (-2.9, -0.37)	Fixed	——	119	2	Low
			Abnormal chest X-ray	RR = 0.29 (0.15, 0.56)	Random	——	126	2	Low
			Average total dosage of hormone (mg)	RR = -770.45 (-1798.47,257.58)	Random	99.20%	109	2	Low
			Daily average total dosage of hormone (mg)	RR = -54.13 (-120.63, 12.38)	Random	——	126	2	Low
			Recovery time of chest X-ray (d)	MD = -2.27 (-3.16, -1.39)	Fixed	——	175	2	Low
			Secondary fungal infection incidence rate (%)	RR = 0.35 (0.14, 0.90)	Random	——	128	2	Low
Zhao 2004	SARS	Traditional Chinese medicine + western medicine vs Western medicine	Mortality (%)	OR = 0.32 (0.14, 0.71)	Random	9.80%	333	4	Low
			Complications caused by hormone use (%)	OR = 0.29 (0.13, 0.65)	Random	0%	33	3	Low
			Time of heat removal (d)	MD = -1.17 (-1.83, -0.5)	Fixed	11.00%	——	5	Low
			Absorption time of lung shadow on chest X-ray	MD = 0.63 (-1.33, 2.59)	Fixed	0%	——	——	Low
			Absorption ratio of lung shadow on chest X-ray	OR = 2.16 (1.22, 3.84)	Random	——	——	——	Low
			Remission time of lower respiratory tract infection	MD = -1.47 (-1.96, -0.98)	Fixed	53.40%	——	——	Low
			Average total dosage of hormone (mg)	MD = -207.19 (-334.98, -69.00)	Fixed	——	——	——	Very Low
			Average time of hormone use (d)	MD = -1.67 (-3.3, -0.03)	Fixed	——	——	——	Low
Pan 2014	H1N1	Chinese patent medicine vs Western medicine	Fever duration(d)	MD = -4.65 (-8.91, -0.38)	Fixed	71.8%	——	5	Low
			Cough duration (d)	MD = -9.79 (-14.61, -4.98)	Fixed	11.2%	320	4	Low
			Sore throat duration (d)	MD = -13.01 (-21.76, -4.27)	Fixed	87.1%	321	4	Low
			Physical pain time (d)	MD = -16.68 (-32.33, -1.03)	Fixed	89.7%	137	3	Very Low
			Nucleic acid negative conversion time (H)	MD = -0.24 (-4.97, 4.31)	Fixed	49.6%	——	5	Low
Jin 2018	Tuberculosis	Traditional Chinese medicine + chemotherapy vs Chemotherapy	Sputum negative conversion rate (%)	RR = 1.30 (1.22, 1.39)	Fixed	35%	2,479	21	High
			Sputum negative conversion rate (%) (after 3 months of treatment)	RR = 1.41 (1.28.1.55)	Fixed	0%	1784	21	High
			Sputum negative conversion rate (%) (after 6months of treatment)	RR = 1.30 (1.22, 1.39)	Fixed	35%	2,479	21	High
			Sputum negative conversion rate (%) (after 9 months of treatment)	RR = 1.35 (1.24, 1.46)	Fixed	40%	1,060	11	High
			Sputum negative conversion rate (%) (after 12 months of treatment)	RR = 1.31 (1.22, 1.42)	Fixed	76%	1,137	12	Moderate
			Sputum negative conversion rate (%) (after 18 months of treatment)	RR = 1.23 (1.14, 1.33)	Fixed	0%	1,461	10	High
			Sputum negative conversion rate (%) (after 24 months of treatment)	RR = 1.32 (1.10, 1.59)	Fixed	0%	252	4	High
			Absorption rate of lesions (%)	RR = 1.08 (1.01, 1.14)	——	——	——	36	Moderate
			Absorption rate of lesions (%) (after 3 months of treatment)	RR = 1.20 (1.10, 1.31)	——	——	——	——	Low
			Absorption rate of lesions (%) (after 6 months of treatment)	RR = 1.08 (1.01, 1.14)	——	59%	——	——	Low
			Absorption rate of lesions (%) (after 9 months of treatment)	RR = 1.29 (1.14, 1.46)	——	——	——	——	Low
			Absorption rate of lesions (%) (after 12 months of treatment)	RR = 1.28 (1.18, 1.40)	——	——	——	——	Low
			Absorption rate of lesions (%) (after 18 months of treatment)	RR = 1.16 (1.09, 1.25)	——	——	——	——	Low
			Absorption rate of lesions (%) after 24 months of treatment)	RR = 1.24 (1.08, 1.43)	——	——	——	——	Low
			Absorption rate of lesions (%) (after 3 months of treatment)	RR = 1.07 (0.85, 1.33)	——	——	——	——	Low
			Absorption rate of lesions (%) (after 6 months of treatment)	RR = 1.11 (0.92, 1.34)	——	——	——	——	Low
			Absorption rate of lesions (%) (after 9 months of treatment)	RR = 1.86 (1.43, 2.42)	——	69%	——	——	Very Low
			Absorption rate of lesions (%) (after 12 months of treatment)	RR = 1.60 (1.25, 2.04)	——	——	——	——	Low
			Absorption rate of lesions (%) (after 18 months of treatment)	RR = 1.16 (1.06, 1.27)	——	——	——	——	Low
			Absorption rate of lesions (%) (after 24 months of treatment)	RR = 1.28 (1.09, 1.51)	——	——	——	——	Low
			Improvement of TCM syndromes (%)	RR = 1.23 (1.17, 1.29)	——	——	——	7	Low
			Improvement of TCM syndromes (%) (after 3 months of treatment)	RR = 1.53 (1.25, 1.87)	——	——	——	——	Low
			Improvement of TCM syndromes (%) (after 6months of treatment)	RR = 1.19 (1.04, 1.36)	——	——	——	——	Low
			Improvement of TCM syndromes (%) (after 9 months of treatment)	RR = 1.19 (1.06, 1.32)	——	>50%	——	——	Low
			Improvement of TCM syndromes (%) (after 12months of treatment)	RR = 1.17 (1.06, 1.29)	——	>50%	——	——	Low
			Improvement of TCM syndromes (%) (after 18 months of treatment)	RR = 1.24 (1.11, 1.37)	——	>50%	——	——	Low
			Improvement of TCM syndromes (%) (after 24 months of treatment)	RR = 1.18 (1.05, 1.32)	——	——	——	——	Low
			Total effective rate (%)	RR = 1.30 (1.21, 1.39)	——	29%	——	10	Moderate
			Adverse reactions incidence rate (%)	RR = 0.65 (0.58, 0.74)	——	——	——	23	Low
Yan 2017	Tuberculosis	Chinese patent medicine + chemotherapy vs Chemotherapy	Sputum negative conversion rate (%) (after 2 months of treatment)	OR = 2.75 (2.10, 3.62)	Fixed	26%	1,316	10	High
			Sputum negative conversion rate (%) (after 3 months of treatment)	OR = 1.70 (1.20, 2.41)	Fixed	0%	914	7	High
			Sputum negative conversion rate (%) (after 6months of treatment)	OR = 1.71 (1.08, 2.70)	Fixed	1%	671	5	High
			Absorption rate of lesions (%) (after 2months of treatment)	OR = 2.19 (1.32, 1.61)	Random	72%	1,424	9	Moderate
			Absorption rate of lesions (%) (after 3 months of treatment)	OR = 1.94 (1.30, 2.90)	Fixed	36%	558	7	Moderate
			Absorption rate of lesions (%) (after 6months of treatment)	OR = 2.06 (1.29, 3.27)	Fixed	43%	457	5	Moderate
			Symptom remission rate (%)	OR = 2.10 (1.52, 2.92)	Fixed	0%	1,128	9	Moderate
			Relief of gastrointestinal tract adverse reactions incidence rate (%)	OR = 0.25 (0.10, 0.62)	Fixed	0%	92	2	Very Low
Yue 2017	Tuberculosis	Coptis chinensis combination Chinese patent medicine + chemotherapy vs Chemotherapy	Sputum negative conversion rate (%)	RR = 1.35 (1.21, 1.50)	Random	82%	3,484	16	Moderate
			Absorption rate of lesions (%)	RR = 1.21 (1.10, 1.32)	Random	88%	2049	15	Moderate
			Void reduction rate (%)	RR = 1.19 (1.08, 1.31)	Random	70%	1,301	11	Moderate
			Improvement rate of clinical symptoms and signs (%)	RR = 1.12 (1.07, 1.16)	Fixed	36%	877	7	Moderate
			Adverse reactions incidence rate (%) (Gastrointestinal reaction incidence rate)	RR = 0.32 (0.24, 0.43)	Fixed	42%	885	6	Moderate
			Adverse reactions incidence rate (%) (Liver function damage incidence rate)	RR = 0.35 (0.25, 0.49)	Fixed	24%	1,044	7	High
			Adverse reactions incidence rate (%) (rash incidence rate)	RR = 0.31 (0.11, 0.87)	Fixed	0%	430	3	High
Wang 2017	Bacterial dysentery	Traditional Chinese medicine + Western medicine vs Western medicine	Total effective rate (%)	OR = 6.87 (3.68, 12.81)	Fixed	0%	1,143	12	High
			Time of heat removal (d)	MD = -1.58 (-1.77, -1.38)	Fixed	92%	454	6	Moderate
			Antidiarrheal time (d)	MD = -1.58 (-1.81, -1.33)	Fixed	94%	429	5	Moderate
Wu 2015	Mumps	Andrographis injection + symptomatic treatment vs Ribavirin + symptomatic treatment	Total effective rate (%) (no antibiotics)	RR = 1.30 (1.12, 1.50)	Fixed	34%	155	3	Low
			Total effective rate (%) (The use of antibiotics was not mentioned)	RR = 1.19 (1.09, 1.31)	Fixed	0%	230	3	Low
			Total effective rate (%)	RR = 1.23 (1.14, 1.33)	Fixed	0%	448	6	Low
			Time of heat removal (no antibiotics)	MD = -1.64 (-1.89, -1.39)	Fixed	40%	446	6	Low
			Time of heat removal (Use of antibiotics)	MD = -0.86 (-1.06, -0.66)	Random	——	60	1	Very Low
			Time of heat removal (The use of antibiotics was not mentioned)	MD = -1.28 (-2.28, -0.29)	Random	99%	312	4	Very Low
			Detumescence time of cheek (no antibiotics)	MD = -2.20 (-2.72, -1.69)	Random	67%	446	6	Low
			Detumescence time of cheek (Use of antibiotics)	MD = -1.60 (-1.87, -1.33)	Random	——	60	1	Very Low
			Detumescence time of cheek (The use of antibiotics was not mentioned)	MD = -2.09 (-3.51, -0.67)	Random	99%	312	4	Very Low
			Detumescence time of cheek	MD = -2.10 (-2.78, -1.41)	Random	97%	818	11	Low
Zhao 2014	Mumps	Traditional Chinese medicine vs Western medicine	Total effective rate (%)	OR = 6.36 (4.85, 8.34)	Fixed	21.6%	2,913	21	Moderate
		Traditional Chinese medicine vs Chinese patent medicine	Total effective rate (%)	OR = 7.93 (3.25, 19.39)	Fixed	0%	432	6	Low
		Traditional Chinese medicine vs Western medicine (Traditional Chinese medicine、western medicine、western medicine)	Total effective rate (%)	OR = 9.94 (5.44, 18.17)	Fixed	20.4%	4,505	6	Moderate
Yu 2020	Hand, foot and mouth disease in children	Ribavirin vs Reduning	Total effective rate (%)	OR = 11.9 (4.64, 3.71)	——	Existence of heterogeneity	1,421	——	Moderate
			Time of heat removal (d)	MD = -2.47 (-4.67, -0.19)	——	Existence of heterogeneity	82	——	Very Low
			Skin rash regression time (d)	MD = -2.83 (-4.25, -1.52)	——	Existence of heterogeneity	160	——	Low
			Healing time of oral ulcer (d)	MD = -1.76 (-3.23, -0.24)	——	Existence of heterogeneity	204	——	Low
			Adverse reactions incidence rate (%)	OR = 0.20 (0.01, 1.64)	——	Existence of heterogeneity	170	——	Low
			Length of stay (d)	MD = -5.88 (-10.80, -0.82)	——	Existence of heterogeneity	——	——	Low
		Ribavirin vs Tanreqing	Total effective rate (%)	OR = 3.21 (0.73, 5.29)	——	Existence of heterogeneity	147	——	Low
			Time of heat removal (d)	MD = -0.99 (-3.03, 1.08)	——	——	63	——	Very Low
			Skin rash regression time (d)	MD = -0.52 (-1.85, 0.88)	——	——	63	——	Very Low
			Healing time of oral ulcer (d)	MD = -1.59 (-3.72, 0.56)	——	——	63	——	Very Low
			Length of stay (d)	MD = -0.76 (-4.04, 2.39)	——	——	63	——	Very Low
		Ribavirin vs Xiyanping	Total effective rate (%)	OR = 6.17 (2.39, 5.72)	——	Existence of heterogeneity	550	——	Low
			Time of heat removal (d)	MD = -1.47 (-2.91, -0.05)	——	Existence of heterogeneity	264	——	Low
			Skin rash regression time (d)	MD = -1.99 (-2.80, -1.18)	——	Existence of heterogeneity	414	——	Low
			Healing time of oral ulcer (d)	MD = -3.58 (-6.52, -0.58)	——	Existence of heterogeneity	——	——	Low
			Adverse reactions incidence rate (%)	OR = 1.29 (0.03, 3.81)	——	Existence of heterogeneity	——	——	Low
			Length of stay (d)	MD = -2.53 (-5.14, 0.18)	——	——	150	——	Low
		Ribavirin vs Yanhuning	Total effective rate (%)	OR = 2.28 (0.72, 5.43)	——	Existence of heterogeneity	86	——	Very Low
			Healing time of oral ulcer (d)	MD = -2.21 (-4.40, -0.07)	——	Existence of heterogeneity	86	——	Very Low
			Length of stay (d)	MD = -1.57 (-5.80, 2.70)	——	——	86	——	Very Low
		Reduning vs Tanreqing	Total effective rate (%)	OR = 3.70 (0.60, 2.24)	——	Existence of heterogeneity	——	——	Very Low
			Time of heat removal (d)	MD = -1.48 (-4.35, 1.39)	——	——	——	——	Very Low
			Skin rash regression time (d)	MD = -2.30 (-4.29, -0.50)	——	Existence of heterogeneity	——	——	Very Low
			Healing time of oral ulcer (d)	MD = -0.17 (-2.80, 2.51)	——	——	——	——	Very Low
			Length of stay (d)	MD = -5.12 (-10.16, 0.27)	——	——	——	——	Very Low
		Reduning vs Xiyanping	Total effective rate (%)	OR = 1.92 (0.58, 7.02)	——	Existence of heterogeneity	64	——	Very Low
			Time of heat removal (d)	MD = -0.98 (-3.14, 1.12)	——	——	64	——	Very Low
			Skin rash regression time (d)	MD = -0.84 (-2.29, 0.45)	——	——	64	——	Very Low
			Healing time of oral ulcer (d)	MD = 1.83 (-1.47, 5.17)	——	——	64	——	Very Low
			Length of stay (d)	MD = -3.38 (-7.44, 0.86)	——	——	64	——	Very Low
			Adverse reactions incidence rate (%)	OR = 0.15 (0.01, 1.82)	——	Existence of heterogeneity	64	——	Very Low
		Reduning vs Yanhuning	Total effective rate (%)	OR = 0.96 (0.02, 9.78)	——	Existence of heterogeneity	——	——	Low
			Healing time of oral ulcer (d)	MD = 0.44 (-2.13, 3.15)	——	——	——	——	Low
			Length of stay (d)	MD = -4.32 (-10.63, 2.44)	——	——	——	——	Low
		Tanreqing vs Yanhuning	Total effective rate (%)	OR = 0.52 (0.11, 2.65)	——	Existence of heterogeneity	——	——	Low
			Time of heat removal (d)	MD = 0.48 (-1.58, 2.54)	——	——	——	——	Low
			Skin rash regression time (d)	MD = 1.46 (0.10, 2.88)	——	Existence of heterogeneity	——	——	Low
			Healing time of oral ulcer (d)	MD = 1.99 (-0.08, 4.07)	——	——	——	——	Low
			Length of stay (d)	MD = 1.76 (-1.57, 4.91)	——	——	——	——	Low
		Tanreqing vs Xiyanping	Total effective rate (%)	OR = 0.25 (0.01, 6.76)	——	Existence of heterogeneity	80	——	Very Low
			Healing time of oral ulcer (d)	MD = 0.62 (-2.35, 3.66)	——	——	80	——	Very Low
			Length of stay (d)	MD = 0.82 (-4.43, 6.14)	——	——	80	——	Very Low
		Xiyanping vs Yanhuning	Total effective rate (%)	OR = 0.50 (0.01, 1.83)	——	Existence of heterogeneity	——	——	Low
			Healing time of oral ulcer (d)	MD = -1.37 (-5.00, 2.32)	——	——	——	——	Low
			Length of stay (d)	MD = -0.94 (-5.84, 4.10)	——	——	——	——	Low
Yang 2020	Hand, foot and mouth disease in children	Chinese patent medicine/Chinese patent medicine + Western medicine vs Western medicine	Total effective rate (%)	RR = 1.20 (1.16, 1.23)	Fixed	45%	3,311	23	Moderate
			Time of heat removal (d)	MD = -1.20 (-1.44, -0.95)	Random	94%	2,708	19	Low
			Herpes disappearance time (d)	MD = -1.78 (-2.10, -1.46)	Random	95%	2,743	19	Low
			Healing time of oral ulcer (d)	MD = -1.45 (-1.62, -1.27)	Random	95%	553	7	Low
			Total duration of disease (d)	MD = -2.22 (-2.39, -2.04)	Random	76%	943	9	Low
			Adverse reactions incidence rate (%)	RR = 1.16 (0.79, 1.70)	Fixed	22%	92	16	Low
Xiong et al. (2019)	Hand, foot and mouth disease in children	Tanreqing + conventional therapy vs Conventional therapy of western medicine	Total effective rate (%)	OR = 2.88 (1.62, 5.10)	Fixed	——	400	3	Low
		Xiyanping injection、Reduning injection/Xiyanping injection、Reduning injection + traditional treatment of western medicine vs Traditional treatment of western medicine	Time of rash regression (H)	MD = -29.57 (-47.18, -11.95)	Random	98%	1,029	9	Low
		Xiyanping injection/Reduning injection vs Conventional therapy of western medicine	Time of rash regression (H) (Traditional Chinese medicine group vs western medicine group)	MD = -27.20 (-50.35, -4.04)	Random	98%	691	5	Low
		Xiyanping injection/Reduning injection + traditional treatment of western medicine vs Traditional treatment of western medicine	Time of rash regression (H) (Integrated traditional Chinese and Western medicine group vs western Medicine group)	MD = -29.57 (-47.28, -11.85)	Random	98%	338	4	Low
		Xiyanping injection、Reduning injection/Xiyanping injection、Reduning injection + conventional therapy of western medicine vs Conventional therapy of western medicine	Antipyretic onset time (H)	MD = -8.10 (-11.77, -4.42)	Fixed	2%	162	4	Low
		Xiyanping injection/Reduning injection vs Traditional treatment of western medicine	Antipyretic onset time (H) (Traditional Chinese medicine group vs western Medicine group)	MD = -9.77 (-18.48, -1.06)	Random	51%	81	2	Very Low
		Xiyanping injection/Reduning injection + Traditional treatment of western medicine vs Traditional treatment of western medicine	Antipyretic onset time (H) (Integrated traditional Chinese and Western medicine group vs Western medicine group)	MD = -7.86 (-13.26, -2.47)	Random	0%	79	2	Very Low
		Xiyanping injection/Reduning injection + Traditional treatment of western medicine vs Traditional treatment of western medicine	Time of heat removal (h) (Subgroup analysis was performed according to the combination of western medicine)	MD = -16.63 (-22.68, -10.59)	Random	98%	1,320	10	Moderate
		Xiyanping injection/Reduning injection/Tanreqing injection vs Traditional treatment of western medicine	Time of heat removal (h) (Subgroup analysis according to the combination of western medicine, traditional Chinese medicine group vs Western medicine group)	MD = -21.91 (-33.61, -10.22)	Random	84%	445	4	Moderate
		Xiyanping injection/Reduning injection/Tanreqing injection + Traditional treatment of western medicine vs Traditional treatment of western medicine	Time of heat removal (h) (Subgroup analysis was performed according to the combined use of western medicine, and the combination group of western medicine and Chinese medicine was compared with the western medicine group)	MD = -13.51 (-21.24, -5.77)	Random	98%	875	5	Low
		Xiyanping injection, Reduning injection, Tanreqing injection	Time of heat removal (h) (Subgroup analysis by traditional Chinese medicine injection)	MD = -18.26 (-27.34, -9.17)	Random	89%	1,326	8	Low
		Tanreqing injection/Tanreqing injection + Traditional treatment of western medicine vs Traditional treatment of western medicine	Time of heat removal (h) (Subgroup analysis according to traditional Chinese medicine injection variety, Tanreqing)	MD = -2.30 (-17.17, 12.56)	Random	81%	323	2	Low
		Xiyanping injection/Xiyanping injection + Traditional treatment of western medicine vs Traditional treatment of western medicine	Time of heat removal (h) (Subgroup analysis by traditional Chinese medicine injection, Xiyanping)	MD = -12.02 (-15.47, -8.56)	Random	0	413	4	Low
		Reduning injection/Reduning injection + Traditional treatment of western medicine vs Traditional treatment of western medicine	Time of heat removal (h) (Subgroup analysis by traditional Chinese medicine injection, Reduning)	MD = -30.48 (-51.95, -9.01)	Random	91%	590	5	Low
		Xiyanping injection, Reduning injection/Xiyanping injection, Reduning injection, Tanreqing injection + Traditional treatment of western medicine vs Traditional treatment of western medicine	Conversion rate of severe cases (%)	OR = 0.83 (0.45, 1.53)	Fixed	0%	1,331	8	High
		Xiyanping injection、Reduning injection/Xiyanping injection、Reduning injection、 Tanreqing injection + Traditional treatment of western medicine vs Traditional treatment of western medicine	Adverse reactions incidence rate (%)	OR = 2.37 (0.39, 14.40)	Fixed	0%	1815	10	Moderate
Yu 2020	Hand, foot and mouth disease	Traditional Chinese medicine vs Western medicine treatment/Traditional Chinese medicine	Disappearance rate of other symptoms	OR = 6.54 (3.59.11.90)	Fixed	0%	142	2	Low
			Duration of fever	OR = -1.04 (-1.60, -0.49)	Random	0%	142	2	Low
			Efficiency	——	——	——	3,925	26	——
			Regression time of hand foot rash	——	——	——	2,262	17	——
			Antipyretic time	——	——	——	2086	16	——

——: Not Reported.

#### COVID-19

Six high-quality SRs (Wang S. et al., 2020; Zeng et al., 2020; Zhou L. P. et al., 2021; Zhou F. et al., 2021; Luo et al., 2021; Ouyang et al., 2021) and eight moderate-quality SRs (Liu and Dong, 2021; Fan et al., 2020; Pang et al., 2020; Jin et al., 2020; Sun et al., 2020; Yang M. et al., 2020; Xiong et al., 2020; Gao et al., 2020) evaluated the efficacy and safety of conventional therapy combined with CHM decoction/proprietary CHM drugs and the results all suggested that this combination therapy was better than conventional therapy alone in improving the overall treatment efficiency for COVID-19 patients.

One single high-quality SR including 19 controlled trials (Luo et al., 2021) identified the efficacy and safety of conventional therapy combined with TCM/tonics, the results showed that the combined with TCM/tonics could improve the appearance of pulmonary CT lesions and the nucleic acid conversion rate, improve the alleviation of symptoms such as fever, cough, malaise, reduce hospitalization time and the rate of clinical cases from mild to severe. However, there was no difference in the incidence of adverse events between the treatments.

Specific to *Lianhuaqingwen* Capsule, a proprietary CHM drug, a moderate quality SR involving seven RCTs (Wang S. et al., 2020) identified the CHM combined with conventional therapy vs. conventional therapy to treat the COVID-19 patients, and the results suggested that the CHM combined with conventional therapy could improve the appearance of pulmonary CT lesions, shorten the fever duration and the time in hospital, and reduce the possibility being worsening. As for safety, no adverse events were reported.

One moderate quality SR including 12 RCTs with mild and ordinary COVID-19 patients (Gao et al., 2020) suggested that the combined with CHM decoction/proprietary CHM drugs could reduce the duration of fever, fatigue, and cough, improve the appearance of pulmonary CT lesions and the nucleic acid conversion rate, and reduce the rate of clinical cases from mild to severe. However, another high-quality systematic review (Ouyang et al., 2021) including six RCTs and four cohort studies identified the efficacy and safety of TCM in the treatment of common or mild COVID-19 patients, showing that TCM was superior to the control group in improving efficiency and reducing the duration of fever, but there was no difference in the relief of related symptoms such as fever and malaise and the incidence of adverse effects between the two groups.

One moderate quality SR involving seven RCTs (Fan et al., 2020) identified the CHM combined with conventional therapy vs. conventional therapy to treat the COVID-19 patients ranging from being mild to severe, and the results suggested that the CHM combined with conventional therapy could improve the appearance of pulmonary CT lesions and reduce C-reactive protein. As for safety, no adverse events were reported.

One single moderate-quality SR including three RCTs (Yang M. et al., 2020) evaluated the efficacy and safety of Lianhuaqingwen capsule, and the results suggested that in combination with conventional treatment, they could improve the alleviation of symptoms such as fever, cough, fatigue, and chest tightness, dyspnoea, and loss of appetite in ordinary COVID-19 patients better than conventional treatment alone. Regarding safety, there was no difference in the incidence of adverse events between the treatments.

One high-quality network meta-analysis including five RCTs (Jin et al., 2020) evaluated the efficacy of four CHM prescripts, namely, *Qingfei Touxie Fuzheng* Decoction, *Lianhua Qingwen* Granule, *Lianhua Qingke* Granule, and *Xuebijing* Injections, and the results suggested that the combination of symptomatic and supportive treatment with either one of four prescriptions could better improve the appearance of the lungs on pulmonary CT than symptomatic treatment alone. Among them, the combination of symptomatic and supportive care with *Lianhua Qingke* Granule had the highest surface under the cumulative ranking (SUCRA) value, suggesting it had the highest overall effectiveness.

Two high-quality systematic reviews (Zhou L. P. et al., 2021; Zhou F. et al., 2021) identified the add-on effect of TCM for COVID-19. One included 10 RCTs and the other included 6 RCTs, and both studies suggested that TCM may be an effective auxiliary treatment for COVID-19 patients, which is likely to help improve the main symptoms, such as fever, cough, and fatigue, shorten the hospital stay and reduce disease progression.

#### SARS

Five moderate-quality SRs (Zhang et al., 2004; Zhao et al., 2004; Hao, 2005; Hao et al., 2005; Liu et al., 2005) evaluated the effectiveness of CHM combined with Western medicine for SARS, and the results all suggested that the combination better improved the clinical progression of SARS patients; however, the benefits to specific outcomes varied across SRs.

One moderate-quality SR including eight controlled trials (Liu et al., 2005) suggested that the additional use of CHM reduced the mortality, the incidence of secondary fungal infections in the lungs, shorten the duration of fever, the persisting clinical symptoms and the time for Chest X-ray to return normal appearance. There were no adverse events for the combination treatments.

Another moderate-quality SR including six RCTs with mild-to-sever patients (Zhang et al., 2004) showed that the improvement of the appearance of abnormal chest X-ray shadows was better in the group treated with CHM decoction and conventional medicine than the conventional treatment alone. However, there was no statistical difference in the reduction of mortality, and dose of corticosteroids, and the alleviation of cough and dyspnoea between two groups.

Two other moderate-quality SR (Hao, 2005; Hao et al., 2005) supported the conclusion the combination of CHM and conventional medicine was better in reducing the duration of fever and mortality among the patients with SARS; however, the use of corticosteroids had not been reduced due to the additional use of CHM.

Another moderate-quality SR (Zhao et al., 2004) did not support the benefits to improving Chest X-ray imaging among the SARs patients when CHM was used alongside conventional medicine; it confirmed the superiority of CHM in reducing the duration of fever, mortality dose of corticosteroids and complications due to overuse of corticosteroids as well as improving clinical symptoms.

#### H1N1 Influenza

One moderate-quality SR including five RCTs (Zhao et al., 2014) suggested that the use of *Lianhua Qingwen* Capsule was better at reducing the duration of symptoms such as fever, cough, sore throat, and body pain in H1N1 patients compared with the use of ooseltamivir. However, there was no statistical difference of the time to conversion to nucleic acid negativity between two treatments. Regarding safety, no details of adverse events were reported.

#### Tuberculosis

One moderate-quality SR (Jin et al., 2018) evaluated the efficacy of CHM decoction/proprietary CHM drugs combined with chemotherapy, and the results suggested that the combination better improved the negative conversion rate of sputum bacteria, lesion absorption rate, lung cavity closure rate, clinical symptom improvement rate, and overall effectiveness of patients with multi-drug-resistant tuberculosis over chemotherapy alone. In terms of safety, the incidence of adverse events was more reduced with the combination treatment.

Specifically, a moderate-quality SR including 16 RCTs (Yan and Gao, 2017) suggested that the proprietary CHM drugs *Jiehe* Pills in combination of chemotherapy better improved the rate of sputum conversion and lesion resorption and alleviated clinical symptoms and signs such as cough, haemoptysis, fever, emaciation, fatigue, and night sweats in tuberculosis patients over chemotherapy alone. In terms of safety, the incidence of digestive discomforts was more reduced with the combination treatment. Another moderate-quality SR including 20 RCTs (Yue et al., 2017) evaluated the efficacy of oral proprietary CHM drugs including Astragalus membranaceus in combination with chemotherapy better improved the rate of sputum conversion and lesion resorption, with less adverse events related to digestive discomforts, liver injury and the occurrence of rash.

#### Bacillary Dysentery

One moderate-quality SR (Wang et al., 2017) evaluated the efficacy of the combined use of CHM decoction and Western conventional therapy, and the results suggested that the combination better improved the overall effectiveness and shortened the time to fever and to diarrhoeal alleviation in adults with bacillary dysentery over Western conventional therapy alone; in terms of safety, digestive disorders were observed (intervention: control: 2 cases versus 5 cases).

#### Mumps

One moderate-quality SR including 11 RCTs (Wu et al., 2015) evaluated the effectiveness of the combined use of *Chuanhuning* Injection versus anti-virus pharmacotherapy ribavirin, and the results suggested that the combined use of *Chuanhuning* Injection and routine care better improved the overall effectiveness, shortened the time to fever and cheek swelling reduction, and reduced the occurrence of complications in children with mumps over ribavirin combined with routine care. In terms of safety, no adverse events occurred in the intervention group compared with the control including 4 cases of adverse events.

Another moderate-quality SR (Zhao, 2014) evaluated the effect of treatment with CHM alone, and the results suggested that internal and external treatment with CHM better improved the overall effectiveness, over proprietary CHM drugs alone; the external use of CHM outperformed the oral treatment. For safety, adverse events were observed, but no details were provided for individual groups.

#### Hand-Foot-And-Mouth Disease

A moderate-quality SR (Xiong et al., 2019) evaluated the effectiveness of proprietary CHM injections alone or in combination with conventional treatment, and the results suggested the monotherapy or the adjunct use of CHM injections reduced the time to fever and rash reduction, and improved the overall clinical effectiveness in children with HFMD. However, there was no difference in the incidence of adverse events and severe case conversion rate between treatments.

A moderate-quality SR including 24 RCTs (Yang Z. et al., 2020) evaluated the effectiveness of using oral proprietary CHM drug *Lanqin* Oral Solution in addition to conventional treatment, and the results suggested that the combination treatment better reduced the time to fever and rash reduction and oral ulcer healing and shortened the total duration of illness in children with HFMD. In terms of safety, there was no difference in the incidence of adverse events between treatments.

One moderate-quality SR including 17 RCTs (Yu et al., 2020a) conducted a network meta-analysis of proprietary CHM drugs for HFMD. The results suggested that the *Yanhuning* Injection, *Reduning* Injection, *Xiyanping* injection and *Tanreqing* injection were significantly better than Ribavirin in improving the total clinical effectiveness; as for oral ulcer healing time and hospitalization time, *Xiyanping* and *Reduning* were significantly shorter than ribavirin*;* in terms of safety, *Reduning* and *Xiyanping* were significantly higher than ribavirin.

Another moderate-quality SR (Yu et al., 2020b) conducted a network meta-analysis to identify the effectiveness and safety of *Qingre Jiedu* TCM oral liquid in the treatment of HFMD. They concluded that seven TCM oral liquids, including *Lanqin* oral liquid, *Pudilan* oral liquid, *Yellow Gardenia* liquid, *Fuganlin* oral liquid, *Kangbindu* oral liquid, *Huangqing* oral liquid, and *Shuanghuanglian* oral liquid, had good therapeutic effects in clinical efficacy and recovery time of related symptoms. In the adverse reactions aspect, *Pudilan* oral liquid had the highest clinical safety.

Supplementary 5 detailed the amount of each drug in a polyherbal preparation, and the complete species and drug name of the included SRs.

## Discussion

This study provides a broad review of the efficacy and safety of CHM in the treatment of acute infectious diseases. After a systematic search and screening, we included 46 systematic reviews, and meta-analysis of moderate-to-high-quality showed that CHM alone or in combination with Western medicine was effective in treating acute and emergent respiratory diseases such as COVID-19, H1N1, and SARS in terms of symptom improvement such as fever, cough and dyspnoea, without serious adverse events. When combined with Western medicine, CHM shows potential in improving certain outcomes, such as mortality, but the evidence is not yet sufficient. In addition, some studies showed that CHM combined with Western medicine can also improve some intermediate outcomes including white blood cell count, nucleic acid negativity conversion rate, lung CT improvement rate. The adjunct use of CHM may be accounted for treating children with acute infections such as HFMD, bacillary dysentery and mumps; however, safety should be closely monitored before and after the treatment.

In the treatment of COVID-19, several moderate-to-high quality systematic reviews and meta-analyses (Yang M. et al., 2020; Fan et al., 2020; Gao et al., 2020; Jin et al., 2020; Pang et al., 2020; Wang S. et al., 2020; Sun et al., 2020; Xiong et al., 2020; Zeng et al., 2020; Luo et al., 2021) showed that combination therapy had a good overall efficiency and nucleic acid negativity conversion rate and alleviated disease symptoms and that CHM may effectively control cytokine storms by inhibiting the excessive activation of immune cells and reducing inflammatory cytokines in relieving COVID-19 symptoms. According to the current overview, the most common drug in the SRs included in this study was *Lianhua Qingwen* Capsule*, a* proprietary CHM drug composed of 13 herbs, namely, the dry fruit of F*orsythia suspensa (Thunb.) Vahl*, the dry buds or with blooming flowers of *Lonicera japonica Thunb.*, the dry caudex of *Ephedra sinica Stapf, Ephedra intermedia Schrenk et C.A.Mey. or Ephedra equisetina Bge.*, the dry matured seeds of *Prunus armeniaca L. var.ansu Maxim.*, *Prunus sibirica L. or Prunus mandshurica (Maxim.) Koehne or Prunus armeniaca L., Gypsum Fibrosum*, the dry roots of *Isatis indigotica Fort.*, the dry roots of *Dryopteris crassirhizoma Nakai.,* the dry aboveground part of *Houttuynia cordata Thunb.*, the dry aboveground part of *Pogostemon cablin (Blanco) Benth*, the dry roots of *Rheum palmatum L.*, the dry roots of *Rhodiola crenulate (Hook. f. et Thoms.) H. Ohba*, the fresh stem of *Mentha haplocalyx Briq.*, and the dry roots and rhizomes of *Glycorrhiza uralensis Fisch.*, *Glycorrhiza inflata Bat. or Glycorrhiza glabra L*. Its benefits for people infected by H1N1 virus and SARS-CoV-2 has been determined by randomised, large-sample, controlled clinical trials, and explained by its capacity of anti-inflammation and immunoregulation in pharmacological experiments (Duan et al., 2011; Huang et al., 2020; Hu et al., 2021). However, some important CHM interventions, for which no SRs have been published yet, probably due to the urgency of the fight against the epidemic, have been published as original studies, while drugs for which clinical studies have been conducted including *Xuebijing* Injection, *Xuanfeibaidu* Decoction, *Qinfeipaidu* Decoction, and *Huashibaidu* Decoction (Wang L. et al., 2020; Xiao et al., 2020; Hu et al., 2021). Substantial publications on prospective/retrospective cohort studies for these CHM prescriptions should be included in future updates of SRs on CHM for acute infections.

For other diseases, a moderate-quality systematic review found that CHM combined with Western medicine for epidemic parotitis shortened the time to fever reduction and improved the overall efficiency, with no significant differences in safety. The main modalities of TCM treatment for mumps include both external and internal application, but validation of the efficacy of these regimens is challenging when designing blinded clinical trials. To enhance and promote exploration of this aspect of the study, some objective outcomes can be selected to be measured as much as possible. Additionally, appropriate reporting guidelines can be selected, such as the CONSORT for Non-Pharmacologic Treatment Interventions (Boutron et al., 2017) and the CONSORT for Chinese Herbal Medicine Formulas (Cheng et al., 2017), to enhance the convenience and operability in conducting systematic reviews.

In addition, the systematic reviews included in this study showed that CHM injections improved the overall clinical effectiveness and severe conversion rate, reduced the time to fever and rash remission and the time for healing of oral ulcers, and shortened the total duration of illness in patients with HFMD. However, none of these SRs reported the occurrence of adverse reactions. HFMD is most prevalent in children, who are a vulnerable group, and there are challenges in conducting clinical studies for this population. Overall, the safety of CHM injections, particularly regarding the amounts used, continues to be of concern. When using CHM injections, one needs to determine whether they are worth using, and if so, their safety needs to be monitored closely.

To the best of our knowledge, this study is the first overview to analyse and evaluate CHM for acute infectious diseases. We systematically assessed 46 systematic reviews and meta-analyses to describe the status of CHM in the treatment of acute infectious diseases. However, the systematic reviews and meta-analyses of CHM alone or in combination with Western medicine for acute infectious diseases were generally plagued with several problems. First, many clinical trials and systematic reviews on Chinese medicine for acute infectious diseases have been published, but most of they are lacking rigorous design and strict quality control. Though time is pressed for fighting against public health emergencies, complying with relevant regulations and methodological consensuses such as *“Best practice in research–overcoming common challenges in phytopharmacological research”*, is necessary for conducting an ethical and high-quality studies. Theses quality-improving issues should be considered in the future research (Heinrich et al., 2020). Second, we only included studies published in Chinese and English, which may lead to publication bias. Last, we are not able to recommend any specific kind of TCM to be used in public health emergencies as the comparative effectiveness between CHM decoction and Chinese patent medicine is to be determined in future studies.

In general, the clinical applicability of existing SRs on the treatment of acute infectious diseases in CHM is not good, and it is suggested that future studies should focus on the staging and typing of diseases, the type of drugs used, and the singularity of interventions. Second, the reporting of outcomes of these systematic reviews is not standardized, and references can be made to the core set of outcomes in TCM for reporting, such as the COVID-19 core outcome set (COS) (Jin et al., 2020; Qiu et al., 2020). In addition, the low quality of reviews can be addressed by strictly following the standards of PRISMA 2020 (Page et al., 2021) and AMSTAR 2 (Shea et al., 2017) when producing future systematic reviews, thus improving the overall quality in the field. Last but not the least, the precise and appropriate use of botanical scientific nomenclature in CHM SRs is further required to avoid ambiguities and error (Rivera et al., 2014).

Although PHEs are a worldwide issue, China has achieved excellent results by applying CHM and Western medicine. For countries that use traditional medicine, there should be more benefits from applying the wisdom of traditional medicine, especially when there is no drug treatment for new and emergency infectious diseases. Moreover, the richness of traditional medicine may also be a source for developing new drugs for emergency infectious diseases, and it would be worthwhile to conduct in-depth research on drugs with a long history of application and clinical effectiveness. However, due to lack of rigorous regulation, the efficacy, safety and quality of some CHM products need to be proved by more high quality, large sample, unbiased randomized trials.

## Conclusion

Overall, CHM, both decoction and Chinese patent medicine, used alone or in combination with conventional medicine may offer potential benefits to relieving symptoms of people with acute respiratory infections. Full reporting of disease typing, staging, and severity, and intervention details is further required for a better evidence translation to the responses for PHE. Future CHM research should focus mainly on the specific aspects of respiratory infections such as its single use for mild infections, and the adjunct administration for sever infections, and individual CHM prescriptions for well-selected outcomes should be prioritized.

## Data Availability

The original contributions presented in the study are included in the article/Supplementary Material, further inquiries can be directed to the corresponding authors.

## Abbreviations

AMSTAR 2, A MeaSurement Tool to Assess systematic Reviews 2; CBM, Chinese Biomedical Literature database; CD4, Cluster of differentiation 4; CHM, Chinese herbal medicine; COS, Core Outcome Set; CNKI, China National Knowledge Infrastructure; COVID-19, Coronavirus disease 2019; CONSORT, Consolidated Standards of Reporting Trials; H1N1, Influenza A H1N1 influenza; HFMD, Hand-foot-and-mouth disease; IL-6 level, Interleukin-6 level; IFN-α, Interferon-alpha; MD, Mean difference; OR, Odds ratio; PHE, Public health emergency; PRISMA, Preferred Reporting Items for Systematic Reviews and Meta-Analyses; RCT, Randomised controlled trial; RD, Rate difference, Risk difference; RoB, Risk of Bias; RR, Relative risk; SARS, Severe Acute Respiratory Syndrome; SMD, Standardized mean difference; SR, Systematic review; SUCRA, Surface under the cumulative ranking; TCM, Traditional Chinese Medicine.
