# Supplementary material for: Clinical Evidence on the Use of Chinese Herbal Medicine for Acute Infectious Diseases: An Overview of Systematic Reviews
Source: Front Pharmacol. 2022 Feb 25;13:752978. doi: 10.3389/fphar.2022.752978 (PMC8914111; doi:10.3389/fphar.2022.752978)
Supplement: Supplementary file 2 [file Table1.DOC]

**Annex 1 Search Strategy**

**Medline (via PubMed)**

#1 “Chinese Medicine” [ti/ab]

#2 “traditional medicine” [ti/ab]

#3 “Chinese herbal” [ti/ab]

#4 “traditional therapy” [ti/ab]

#5 “traditional treatment” [ti/ab]

#6 OR#1-#5

#7 "Public Health Emergenc*"[ti/ab]

#8 "emergency medicine*"[ti/ab]

#9 "public health emergency preparedness"[ti/ab]

#10 “emergency department*"[ti/ab]

#11 “emergency preparedness"[ti/ab]

#12 “emerging infection*”[ti/ab]

#13 “health emergency” [ti/ab]

#14 “Public Health Surveillance” [ti/ab]

#15 “Disease Outbreaks” [ti/ab]

#16 OR#7-#15

#17 #6 AND #16

CNKI (In Chinese)

#1 中医[主题]

#2 中药[主题]

#3 中医药[主题]

#4 传统医学[主题]

#5 传统疗法[主题]

#6 OR#1-#5

#7 “突发公共卫生事件”[主题]

#8 “公共卫生应急”[主题]

#9 “疫情”[主题]

#10 “灾情”[主题]

#11 “抗疫”[主题]

#12 “抗灾”[主题]

#13 “卫生应急”[主题]

#14 “应急管理”[主题]

#15 “预防医学”[主题]

#16 “重大传染病”[主题]

#17 “突发传染病”[主题]

#18 OR#7-#17

#19 #6 AND #18
