# Supplementary material for: Clinical Evidence on the Use of Chinese Herbal Medicine for Acute Infectious Diseases: An Overview of Systematic Reviews
Source: Front Pharmacol. 2022 Feb 25;13:752978. doi: 10.3389/fphar.2022.752978 (PMC8914111; doi:10.3389/fphar.2022.752978)
Supplement: Supplementary file 3 [file Table5.DOCX]

**A summary of scientific nomenclature for CHM ingredients**

| **Formula name** | **Species, concentration** | **Being included to Chinese pharmacopoeia (Y/N）** |
| --- | --- | --- |
| Qingkailing injection (Liu 2004) | 1. The shells of Hyriopsis cumingii (Lea), Cristaria plicata (Leach) or Pteria martensii (Dunker); 2. The dry mature fruit of Gardenia jasminoides Ellis ; 3. The horn of Bubalus bubalis Linnaeus ; 4. The dry roots of Isatis indigotica Fort.; 5. The dry roots of Scutellaria baicalensis Georgi; 6. The dry buds or with blooming flowers of Lonicera japonica Thunb. | Y |
| Xuesaitong injection (Liu 2004) | The dry roots and rhizomes of Panax notoginseng (Burk.) F. H. Chen | N |
| Compound herbs No. 1 (Liu 2012) | 1. The dry roots of Panax quinquefolium L.6g; 2. The dry root tubers of Ophiopogon japonicus (L.f) Ker-Gawl.12g; 3. The Dry ripe fruit of Schisandra chinensis (Turcz.) Baill.6g; 4. The dry roots of Astragalus membranaceus (Fisch.) Bge.var.mongholicus (Bge.) Hsiao or Astragalus membranaceus (Fisch.) Bge.18g; 5. The dry rhizome of Polygonatum odoratum (Mill.) Druce 12g; 6. The dry roots of Trichosanthes kirilowii Maxim. or Trichosanthes rosthornii Harms 12g; 7. The dry rhizome of Atractylodes macrocephala Koidz.9g; 8. The dry sclerotia of Poria cocos (Schw.) Wolf 12g; 9. The Dry leaves of Morus albaL.12g; 10. The dry roots of Angelica sinensis (Oliv.) Diels 9g; 11. The Dry roots of Paeonia lactiflora Pall.12g; 12. The dry roots of Ligusticum chuanxiong Hort.12g; 13. The dry leaves of Nelumbo nucifera Gaertn.10g; 14. Talcum [Mg_3_(Si_4_O_10_)(OH)_2_]； 15. The dry roots and rhizomes of Glycorrhiza uralensis Fisch., Glycorrhiza inflata Bat. or Glycorrhiza glabra L. | N |
| Compound herbs No. 2 (Liu 2012) | 1. The dry roots of Astragalus membranaceus (Fisch.) Bge.var.mongholicus (Bge.) Hsiao or Astragalus membranaceus (Fisch.) Bge.30g; 2. The dry roots of Codonopsis pilosula (Franch.)Nannf.,Codonopsis pilosula Nannf.var.modesta (Nannf.) L.T.Shen or Codonopsis tangshenOliv.15g; 3. The dry rhizome of Atractylodes macrocephala Koidz.15g; 4. The dry sclerotia Poria cocos (Schw.) Wolf. 15g; 5. The dry roots of Bupleurum chinense DC. or Bupleurum scorzonerifolium Willd.9g; 6. The Dry roots of Paeonia lactiflora Pall.12g; 7. The dry roots of Angelica sinensis (Oliv.) Diels 9g; 8. The dry roots of Aucklandia lappa Decne.12g; 9. The dry mature fruit of Amomum villosum Lour., Amomum villosum Lour.var.xanthioides T.L.Wu et Senjen or Amomum longiligulare T.L.Wu 6g; 10. The dry and mature pericarp of Citrus reticulata Blanco and its cultivated varieties in Rutaceae 12g; 11. The dry tubers of Pinellia ternata (Thunb.) Breit.9g; 12. The dry aboveground part of Pogostemon cablin (Blanco) Benth.10g; 13. The mature fruit of Hordeum vulgare L.3g; 14. The dry ripe fruit of Crataegus pinnatifida Bge. var. Major N. E. Br. or Crataegus pinnatifida Bge.3g; 15. Massa Medicata Fermentata. 3g; | N |
| Compound herbs No. 3 (Liu 2012) | 1. The dry roots of Panax quinquefolium L.3g; 2. The dry roots of Glehnia littoralis Fr. Schmidtex Miq.15g; 3. The dry root tubers of Ophiopogon japonicus (L.f) Ker-Gawl.12g; 4. The dry roots of Bupleurum chinense DC. or Bupleurum scorzonerifolium Willd.9g; 5. The dry roots of Astragalus membranaceus (Fisch.) Bge.var.mongholicus (Bge.) Hsiao or Astragalus membranaceus (Fisch.) Bge.12g; 6. The dry leaves of Morus albaL.15g; 7. The dry root bark of Morus alba L.15g; 8. The dry root bark of Lycium chinense Mill. or Lycium barbarum L.12g; 9. The Dry aboveground part of Artemisia annua L.15g; 10. The fresh or dry rhizomes of Phragmites communis Trin.15g; 11. The dry rhizome of Imperata cylindrica Beauv.var.major (Nees) C.E.Hubb.15g; 12. The dry roots of Angelica sinensis (Oliv.) Diels 9g; 13. The Dry roots of Paeonia lactiflora Pall.12g; 14. The dry tubers of Pinellia ternata (Thunb.) Breit.9g; 15. The mature fruit of Hordeum vulgare L.15g; 16. The mature fruit of Setaria italica (L.) Beauv.15g | N |
| National drug No. 2 (Liu 2012) | 1. Gypsum Fibrosum (CaSO4·2H2O); 2. The dry roots of Astragalus membranaceus (Fisch.) Bge.var.mongholicus (Bge.) Hsiao or Astragalus membranaceus (Fisch.) Bge. 3. The dry roots of Arnebia euchroma (Royle) Johnst. or Arnebia guttata Bunge | N |
| National drug No. 3 (Liu 2012) | 1. The fresh or dry root tubers of Rehmannia glutinosa Libosch.; 2. The dry roots of Scrophularia ningpoensis Hemsl.; 3. The dry buds or with blooming flowers of Lonicera japonica Thunb. | N |
| National drug No. 4 (Liu 2012) | 1. The dry root tubers of Pseudostellaria heterophylla (Miq.) Pax ex Pax et Hoffm.; 2. The dry roots of Glehnia littoralis Fr. Schmidtex Miq.; 3. The dry roots of Codonopsis pilosula (Franch.) Nannf., Codonopsis pilosula Nannf.var.modesta (Nannf.) L.T.Shen or Codonopsis tangshenOliv. | N |
| Kangfeidian No. 1 (Liu 2012) | 1. Gypsum Fibrosum (CaSO4·2H2O)45g; 2. The dry roots of Bupleurum chinense DC. or Bupleurum scorzonerifolium Willd.15g; 3. The dry rhizome of Anemarrhena asphodeloides Bge.10g; 4. The dry scales of Fritillaria thunbergii Miq.10g; 5. The dry roots of Scutellaria baicalensis Georgi 15g; 6. The Dry aboveground part of Artemisia annua L.15g; 7. The dry root bark of Paeonia suffruticosa Andr.10g; 8. The dry roots of Paeonia lactiflora Pall. or Paeonia veitchii Lynch 12g; 9. The dry fruit of Forsythia suspensa (Thunb.) Vahl 15g; 10. The dry mature pulp of Cornus officinalis Sieb. et Zucc.30g; 11. The dry rhizome of Atractylodes lancea (Thunb.) DC. or Atractylodes chinensis (DC .) Koidz.15g; 12. The dry aboveground part of Pogostemon cablin (Blanco) Benth.10g; 13. The dry mature kernel of Coix lacryma-jobi L.var.ma-yuen (Roman.) Stapf 15g; 14. The dry mature seeds of Prunus armeniaca L.var.ansu Maxim.,Prunus sibirica L.,Prunus mandshurica (Maxim.) Koehne or Prunus armeniaca L.10g | N |
| Kangfeidian No. 2 (Liu 2012) | 1. The dry roots of Astragalus membranaceus (Fisch.) Bge.var.mongholicus (Bge.) Hsiao or Astragalus membranaceus (Fisch.) Bge.15g; 2. The Dry aboveground part of Artemisia annua L.15g; 3. The dry mature fruit of Trichosanthes kirilowii Maxim. or Trichosanthes rosthornii Harms 30g; 4. The dry roots of Codonopsis pilosula (Franch.) Nannf.、Codonopsis pilosula Nannf.var.modesta (Nannf.) L.T.Shen or Codonopsis tangshenOliv.15g; 5. The dry head of Inula japonica Thunb. or Inula britannica L.10g; 6. The dry root tubers of Curcuma wenyujin Y. H. Chen et C. Ling,Curcuma Longa L. ,Curcuma kwangsiensis S. g. Lee et C. F. Liang or Curcuma phaeocaulis Vai.10g; 7. The dry rhizome of Acorus tatarinowii Schott 10g; 8. The dry rhizome of Dioscorea septemloba Thunbt or Dioscorea hypoglauca Palibin 12g; 9. silkworm shit 15g; 10. The dry rhizome of Atractylodes lancea (Thunb.) DC. or Atractylodes chinensis (DC.) Koidz.15g; 11. The dry rhizome of Atractylodes macrocephala Koidz.15g; 12. The dry sclerotia of Polyporus umbellatus (Pers.) Fries 15g; 13. The dry sclerotia of Poria cocos (Schw.) Wolf 15g; 14. The dry mature kernel of Coix lacryma-jobi L.var.ma-yuen(Roman.) Stapf 15g; 15. The dry mature seeds of Prunus armeniaca L.var.ansu Maxim.,Prunus sibirica L.,Prunus mandshurica (Maxim.) Koehne or Prunus armeniaca L.10g; 16. The dry mature seeds of Plantago asiatica L. or Plantago depressa Willd.10g; 17. The dry mature pulp of Cornus officinalis Sieb. et Zucc.30g | N |
| Kangfeidian No. 3 (Liu 2012) | 1. The dry roots of Panax quinquefolium L.30g; 2. The dry roots of Astragalus membranaceus (Fisch.) Bge.var.mongholicus (Bge.) Hsiao or Astragalus membranaceus (Fisch.) Bge.30g; 3. The dry mature pulp of Cornus officinalis Sieb. et Zucc.30g; 4. The dry root tubers of Ophiopogon japonicus (L.f) Ker-Gawl.15g; 5. The dry rhizome of Anemarrhena asphodeloides Bge.10g; 6. The dry scales of Fritillaria thunbergii Miq.10g; 7. Bombyx Batryticatus.30g; 8. The dry fruit of Forsythia suspensa (Thunb.) Vahl 15g; 9. The dry roots of Codonopsis pilosula (Franch.)Nannf., Codonopsis pilosula Nannf.var.modesta（Nannf.）L.T.Shen or Codonopsis tangshenOliv.15g; 10. Citri Reticulatae Pericarpium Viride 12g; 11. Bombyx mori L.15g; 12. The dry mature kernel of Coix lacryma-jobi L.var.ma-yuen (Roman.) Stapf 15g; 13. The dry sclerotia of Polyporus umbellatus (Pers.) Fries 15g; 14. The dry sclerotia of Poria cocos (Schw.) Wolf 15g; 15. The dry mature fruit of Trichosanthes kirilowii Maxim. or Trichosanthes rosthornii Harms 30g; 16. The dry roots and rhizomes of Aster tataricus L. f.15g | N |
| Compound Herbal formulas: When the participants had a fever, the following formula was used (Liu 2012) | 1. The dry straw of Ephedra sinica Stapf,Ephedra intermedia Schrenk et C.A.Mey. or Ephedra equisetina Bge.5g; 2. The dry mature seeds of Prunus armeniaca L.var.ansu Maxim.,Prunus sibirica L.,Prunus mandshurica (Maxim.) Koehne or Prunus armeniaca L.12g; 3. Gypsum Fibrosum 45g; 4. The dry rhizome of Anemarrhena asphodeloides Bge.10g; 5. The dry buds or with blooming flowers of Lonicera japonica Thunb.15g; 6. The dry fruit of Forsythia suspensa (Thunb.) Vahl 12g; 7. The dry mature fruit of Gardenia jasminoides Ellis 12g; 8. The dry roots of Scutellaria baicalensis Georgi 12g; 9. The dry leaves of Perilla frutescens (L.) Britt.10g; 10. The dry aboveground part of Artemisia scoparia Waldst.etKit. or Artemisia capillaris Thunb.15g; 11. The dry roots of Pueraria lobata (Willd.) Ohwi 15g; 12. The dry root tubers of Pseudostellaria heterophylla (Miq.) Pax ex Pax et Hoffm.15g | N |
| Compound Herbal formulas:When the participants had a cough, the following formula was used (Liu 2012) | 1. The dry roots of Panax quinquefolium L.15g; 2. The dry root tubers of Ophiopogon japonicus (L.f )Ker-Gawl.15g; 3. The Dry ripe fruit of Schisandra chinensis(Turcz. )Baill.10g; 4. The dry mature pulp of Cornus officinalis Sieb. et Zucc.12g; 5. The dry mature seeds of Descurainia Sophia (L.)Webb. ex Prantl. or Lepidium apetalum Willd.15g; 6. The dry roots and rhizomes of Aster tataricus L. f.15g; 7. The dry branches and leaves of Platycladus orientalis (L.)Franco 12g; 8. Pheretima 12g; 9. The dry roots and rhizomes of Salvia miltiorrhiza Bge.12g; 10. The dry buds or with blooming flowers of Lonicera japonica Thunb.8g; 11. The dry roots of Scutellaria baicalensis Georgi 10g; 12. The dry mature pericarp of Trichosanthes kirilowii Maxim. or Trichosanthes rosthornii Harms 15g; 13. The dry roots of Pueraria lobata (Willd.) Ohwi | N |
| Compound Herbal formulas: When the participants were in the convalescence stage, the following formula was used (Liu 2012) | 1. The dry root tubers of Pseudostellaria heterophylla (Miq.) Pax ex Pax et Hoffm.15g; 2. The dry root tubers of Ophiopogon japonicus (L.f) Ker-Gawl.15g; 3. The dry roots of Glehnia littoralis Fr. Schmidtex Miq.15; 4. The dry rhizome of Atractylodes macrocephala Koidz.15g; 5. The Dry branches and leaves of Platycladus orientalis (L.) Franco 6. The dry mature fruit of Amomum villosum Lour.,Amomum villosum Lour.var.xanthioides T.L.Wu et Senjen or Amomum longiligulare T.L.Wu 6g; 7. The mature fruit of Hordeum vulgare L.30g; 8. The dry ripe fruit of Crataegus pinnatifida Bge. var. Major N. E. Br. or Crataegus pinnatifida Bge.30g; 9. Massa Medicata Fermentata30g; 10. The dry roots of Astragalus membranaceus (Fisch.) Bge.var.mongholicus (Bge.) Hsiao or Astragalus membranaceus (Fisch.) Bge.15g; 11. The dry roots of Pueraria lobata (Willd.) Ohwi 15g; 12. The dry roots and rhizomes of Salvia miltiorrhiza Bge.15g; 13. The dry and mature pericarp of Citrus reticulata Blanco and its cultivated varieties in Rutaceae 6g; 14. The dry rhizome of Polygonatum kingianum Coll.et Hemsl.,Polygonatum sibiricum Red. Or Polygonatum cyrtonema Hua 15g | N |
| Chuanghuning injection (Liu 2012) | The dry aboveground part of Andrographis paniculata (Burm.f.) Nees | N |
| Shengmai injection (Liu 2012) | 1. The dry roots and rhizomes of Panax ginseng C.A.Mey.; 2. The dry root tubers of Ophiopogon japonicus (L.f) Ker-Gawl.; 3. The Dry ripe fruit of Schisandra chinensis (Turcz.) Baill. | N |
| Compound Chinese herbs (x) Yi Qi Yang Ying recipe (Liu 2012) | 1. The dry roots of Panax quinquefolium L. 2. The dry root tubers of Ophiopogon japonicus (L.f) Ker-Gawl.; 3. The Dry ripe fruit of Schisandra chinensis (Turcz.) Baill.; 4. The dry roots of Astragalus membranaceus (Fisch.) Bge.var.mongholicus (Bge.) Hsiao or Astragalus membranaceus (Fisch.) Bge.; 5. The dry rhizome of Polygonatum odoratum (Mill.) Druce; 6. The dry roots of Trichosanthes kirilowii Maxim. or Trichosanthes rosthornii Harms | N |
| Compound Chinese herbs (x) Bu Fei Jian Pi recipe (Liu 2012) | 1. The dry roots of Astragalus membranaceus (Fisch.) Bge.var.mongholicus (Bge.) Hsiao or Astragalus membranaceus (Fisch.) Bge.; 2. The dry roots of Codonopsis pilosula (Franch.)Nannf., Codonopsis pilosula Nannf.var.modesta（Nannf.）L.T.Shen or Codonopsis tangshenOliv.; 3. The dry rhizome of Atractylodes macrocephala Koidz.; 4. The dry sclerotia of Poria cocos (Schw.) Wolf; 5. The dry roots of Bupleurum chinense DC. or Bupleurum scorzonerifolium Willd.; 6. The Dry roots of Paeonia lactiflora Pall.; 7. The dry roots of Angelica sinensis (Oliv.) Diels | N |
| Compound Chinese herbs (x) Yang Yin Qing Re recipe (Liu 2012) | 1. The dry roots of Panax quinquefolium L.; 2. The dry roots of Glehnia littoralis Fr. Schmidtex Miq.； 3. The dry root tubers of Ophiopogon japonicus (L.f) Ker-Gawl.； 4. The dry roots of Bupleurum chinense DC. or Bupleurum scorzonerifolium Willd.； 5. The dry roots of Astragalus membranaceus (Fisch.) Bge.var.mongholicus (Bge.) Hsiao or Astragalus membranaceus (Fisch.) Bge.； 6. The dry rhizome of Dioscorea opposita Thunb.； 7. The dry root bark of Lycium chinense Mill. or Lycium barbarum L.； 8. The Dry aboveground part of Artemisia annua L. | N |
| Compound Chinese herbs (z) Yi Qi Yang Ying recipe (Liu 2012) | 1. The dry root tubers of Pseudostellaria heterophylla (Miq.) Pax ex Pax et Hoffm.30g; 2. The dry root tubers of Ophiopogon japonicus (L.f) Ker-Gawl.12g; 3. The Dry ripe fruit of Schisandra chinensis (Turcz.) Baill.6g; 4. The dry roots of Astragalus membranaceus (Fisch.) Bge.var.mongholicus（Bge.）Hsiao or Astragalus membranaceus (Fisch.) Bge.15g; 5. The dry roots of Trichosanthes kirilowii Maxim. or Trichosanthes rosthornii Harms 12g; 6. The dry sclerotia of Poria cocos (Schw.) Wolf 12g; 7. The dry roots of Ligusticum chuanxiong Hort.12g; 8. The dry roots of Angelica sinensis (Oliv.) Diels 9g; 9. The Dry roots of Paeonia lactiflora Pall.9g; 10. The dry rhizome of Atractylodes macrocephala Koidz.15g; 11. The dry roots and rhizomes of Glycorrhiza uralensis Fisch., Glycorrhiza inflata Bat. or Glycorrhiza glabra L.9g; 12. The dry roots of Bupleurum chinense DC. or Bupleurum scorzonerifolium Willd.12g; 13. Talcum； 14. The dry roots and rhizomes of Glycorrhiza uralensis Fisch., Glycorrhiza inflata Bat. or Glycorrhiza glabra L. | N |
| Compound Chinese herbs (z) Bu Fei Jian Pi recipe (Liu 2012) | 1. The dry roots of Astragalus membranaceus(Fisch.) Bge.var.mongholicus (Bge. )Hsiao or Astragalus membranaceus(Fisch. )Bge.20g; 2. The dry roots of Codonopsis pilosula (Franch.)Nannf., Codonopsis pilosula Nannf.var.modesta (Nannf.) L.T.Shen or Codonopsis tangshenOliv.15g; 3. The dry rhizome of Atractylodes macrocephala Koidz.15g; 4. The dry sclerotia of Poria cocos (Schw.)Wolf15g,; 5. The dry roots of Angelica sinensis (Oliv.)Diels 9g; 6. The Dry roots of Paeonia lactiflora Pall.12g; 7. The dry roots of Ligusticum chuanxiong Hort.12g; 8. The dry roots of Aucklandia lappa Decne. 12g; 9. Citrus reticulata Blanco 12g; 10. The dry aboveground part of Pogostemon cablin (Blanco)Benth.10g; 11. The mature fruit of Hordeum vulgare L.10g; 12. The dry ripe fruit of Crataegus pinnatifida Bge. var. Major N. E. Br. or Crataegus pinnatifida Bge.10g; 13. Massa Medicata Fermentata.10g; 14. Amomum villosum Lour. Or Amomum villosum Lour.var.xanthioides T.L.Wu et Senjen or Amomum longiligulare T.L.Wu 6g | N |
| Compound Chinese herbs (z) Yang Yin Qing Re recipe (Liu 2012) | 1. The dry root tubers of Pseudostellaria heterophylla (Miq.) Pax ex Pax et Hoffm.15g; 2. The dry mature fruit of Amomum villosum Lour.,Amomum villosum Lour.var.xanthioides T.L.Wu et Senjen or Amomum longiligulare T.L.Wu 15g; 3. The dry root tubers of Ophiopogon japonicus (L.f) Ker-Gawl.12g; 4. The dry roots of Bupleurum chinense DC. or Bupleurum scorzonerifolium Willd.9g; 5. The young leaf of Bupleurum chinense DC. or Bupleurum scorzonerifolium Willd.9g; 6. The dry leaves of Morus albaL.15g; 7. The dry root bark of Morus alba L.15g; 8. The dry roots of Scutellaria baicalensis Georgi 12g; 9. The dry root bark of Lycium chinense Mill. or Lycium barbarum L.12g; 10. The Dry aboveground part of Artemisia annua L.15g; 11. The fresh or dry rhizomes of Phragmites communis Trin.15g； 12. The dry tubers of Pinellia ternata (Thunb.) Breit.9g; 13. The Dry roots of Paeonia lactiflora Pall.12g; 14. The dry roots of Angelica sinensis (Oliv.) Diels 9g; 15. The dry roots of Astragalus membranaceus (Fisch.) Bge.var.mongholicus (Bge.) Hsiao or Astragalus membranaceus (Fisch.) Bge.12g; 16. Talcum 10g； 17. The dry roots and rhizomes of Glycorrhiza uralensis Fisch., Glycorrhiza inflata Bat. or Glycorrhiza glabra L 10g; 18. The dry root bark of Paeonia suffruticosa Andr. 12g | N |
| Yingqing heji (for normal SARS participants) (Liu 2012) | 1. The dry buds or with blooming flowers of Lonicera japonica Thunb.20g; 2. The dry roots of Dryopteris crassirhizoma Nakai 15g; 3. The dry leaf of Isatis indigotica Fort.20g; 4. The dry roots of Pueraria lobata (Willd.) Ohwi 15g; 5. The dry leaf of Perilla frutescens (L.) Britt.12g; 6. The dry roots of Platycodon grandiflorum (Jacq.) A.DC 15g; 7. The dry aboveground part of Pogostemon cablin (Blanco) Benth.15g; 8. The dry roots and rhizomes of Glycorrhiza uralensis Fisch., Glycorrhiza inflata Bat. or Glycorrhiza glabra L.30g | N |
| Compound Chinese herbs (j) Yuxing heji (for severe and acute sever SARS patients) (Liu 2012) | 1. The dry aboveground part of Houttuynia cordata Thunb.45g; 2. The dry roots of Isatis indigotica Fort.45g; 3. The dry roots of Scutellaria baicalensis Georgi 15g; 4. The dry seed of Prunus armeniaca L.var.ansu Maxim. Or Prunus sibirica L.or Prunus mandshurica(Maxim.)Koehne or Prunus armeniaca L. 15g; 5. The dry roots of Bupleurum chinense DC. or Bupleurum scorzonerifolium Willd.15g; 6. The dry aboveground part of Artemisia annua L.15g; 7. The dry aboveground part of Agrimonia pilosa Ledeb.20g; 8. Dried intermediate layer of stalks Bambusa tuldoides Munro or Sinocalamus beecheyanus (Munro)McClure var. Pubescens P.F.Li or Phyllostachys nigra (Lodd. ) Munro var.henonis(Mitf. )Stapf ex Rendle 15g; 9. Gypsum Fibrosum (CaSO4·2H2O) 30g; 10. The dry roots of Anemarrhena asphodeloides Bge. 20g; 11. The dry roots of Pseudostellaria heterophylla (Miq. )Pax ex Pax et Hoffm.20g; 12. The dry roots and rhizomes of Glycorrhiza uralensis Fisch., Glycorrhiza inflata Bat. or Glycorrhiza glabra L.30g | N |
| Compound Chinese herbs (j) Ganqi heji (Liu 2012) | 1. The dry roots of Astragalus membranaceus (Fisch.) Bge.var.mongholicus (Bge. )Hsiao or Astragalus membranaceus (Fisch.) Bge.45g; 2. The dry roots and rhizomes of Glycorrhiza uralensis Fisch., Glycorrhiza inflata Bat. or Glycorrhiza glabra L. 30g,; 3. The dry mature fruit of Prunus persica (L. )Batsch or Prunus davidiana (Carr.) Franch.30g; 4. Trionyx sinensis Wiegmann 30g | N |
| Compound Chinese herbs (j) Kangfeidiang No. 1 granule (Liu 2012) | 1. The dry roots of Dryopteris crassirhizoma Nakai 20g; 2. The dry roots of Bupleurum chinense DC. or Bupleurum scorzonerifolium Willd.10g; 3. The Dry ripe fruit of Schisandra chinensis(Turcz. ) Baill.6g | N |
| Compound Chinese herbs (j) Kangfeidian No. 2 granule (Liu 2012) | 1. The dry buds or with blooming flowers of Lonicera japonica Thunb.10g; 2. The dry roots of Dryopteris crassirhizoma Nakai 10g; 3. The dry leaf of Isatis indigotica Fort.10g | N |
| Compound Chinese herbs (y) 1 (Liu 2012) | 1. Gypsum Fibrosum (CaSO4·2H2O) 30-50g; 2. The dry roots of Anemarrhena asphodeloides Bge. 10g; 3. The dry buds or with blooming flowers of Lonicera japonica Thunb. 30g; 4. The stems, branches of Lonicera japonica Thunb.30g; 5. The dry fruit of Forsythia suspensa (Thunb.) Vahl 10g; 6. The dry roots of Notopterygium incisum Ting ex H. T. Chang or Notopterygium franchetii H. de Boiss.10g; 7. The dry aboveground part of Mentha haplocalyx Briq.10g; 8. The dry roots and rhizomes of Glycorrhiza uralensis Fisch., Glycorrhiza inflata Bat. or Glycorrhiza glabra L.6g; 9. Saiga tatarica Linnaeus 0.3g | N |
| Compound Chinese herbs (y): 2 (Liu 2012) | 1. The dry roots of Astragalus membranaceus (Fisch.) Bge.var.mongholicus (Bge.) Hsiao or Astragalus membranaceus (Fisch.) Bge. 15-30g; 2. Gypsum Fibrosum (CaSO4·2H2O) 30g; 3. Saiga tatarica Linnaeus 0.6g; 4. Bambusa textilis McClure or Schizostachyum chinense Rendle 10g; 5. The dry roots of Salvia miltiorrhiza Bge.15-30g; 6. Panax notoginseng (Burk.) F. H. Chen 3g | N |
| Compound Chinese herbs (y): 3 (Liu 2012) | 1. The dry roots of Pseudostellaria heterophylla (Miq.) Pax ex Pax et Hoffm.15g; 2. The dry roots of Astragalus membranaceus (Fisch.) Bge.var.mongholicus (Bge.) Hsiao or Astragalus membranaceus (Fisch.) Bge.15-20g; 3. The dry roots of Atractylodes lancea (Thunb.) DC. or Atractylodes chinensis (DC.) Koidz.10 g; 4. The dry rhizome of Atractylodes macrocephala Koidz.10 g; 5. The Dried mature seed of Dolichos lablab L.30 g; 6. The Dried mature seed kernel of Coix lacryma-jobi L.var.ma-yuen (Roman.) Stapf 30 g; 7. The dry mature fruit of Trichosanthes kirilowii Maxim.or Trichosanthes rosthornii Harms 10g; 8. The dry mature fruit of Luffa cylindrica (L.) Roem.10g; 9. The dry roots of Salvia miltiorrhiza Bge.30g; 10. The dry leaf of Platycladus orientalis (L.) Franco 10g | N |
| Compound Chinese herbs (ls) (When treatment was in progress, the following recipe was used.) (Liu 2012) | 1. The dry roots of Rhaponticum uniflorum (L.) DC. 15g; 2. The dry fruit of Forsythia suspensa (Thunb.) Vahl 12g; 3. The dry buds or with blooming flowers of Lonicera japonica Thunb. 15g; 4. The dry roots of Scutellaria baicalensis Georgi 10g; 5. The dry aboveground part of Artemisia annua L.15g; 6. CaSO4·2H2O 30g; 7. The dry mature fruit of Trichosanthes kirilowii Maxim.or Trichosanthes rosthornii Harms 15g; 8. The dry roots of Fritillaria thunbergii Miq.15g; 9. The dry mature fruit of Gardenia jasminoides Ellis10g; 10. The dry mature fruit of Plantago asiatica L.or Plantago depressa Willd. 10g; 11. The dry roots of Paeonia lactiflora Pall. Or Paeonia veitchii Lynch 12g | N |
| Compound Chinese herbs (ls) (When patients were at recovery stage, the following recipe was used.) (Liu 2012) | 1. Cordyceps sinensis (BerK.) Sacc. 15g; 2. The dry roots of Pseudostellaria heterophylla (L.) Miq.) Pax ex Pax et Hoffm.20g; 3. The dry roots of Scrophularia ningpoensis Hemsl.12g; 4. The dry roots of Paeonia lactiflora Pall. Or Paeonia veitchii Lynch 12g; 5. The dry roots of Rhaponticum uniflorum (L.) DC.15g; 6. The dry fruit of Forsythia suspensa (Thunb.) Vahl 12g; 7. Panax notoginseng (Burk.) F. H. Chen 3g; 8. The mature fruit of Hordeum vulgare L.10g; 9. The dry ripe fruit of Crataegus pinnatifida Bge. var. Major N. E. Br. or Crataegus pinnatifida Bge.10g; 10. Massa Medicata Fermentata.10 g; 11. The shell of Gardenia jasminoides Ellis 10g; 12. the Dried mature seed kernel of Coix lacryma-jobi L.var.ma-yuen (Roman.) Stapf 30g | N |
| Kangbingdu oral solution (Yan 2020) | 1. The dry roots of Isatis indigotica Fort.; 2. Gypsum Fibrosum (CaSO4·2H2O); 3. The dry roots of Phragmites communis Trin.; 4. The dry roots of Rehmannia glutinosa Libosch; 5. The dry roots of Curcuma wenyujin Y. H. Chen et C. Ling, Curcuma Longa L., Curcuma kwangsiensis S. g. Lee et C. F. Liang or Curcuma phaeocaulis Vai; 6. The dry roots of Anemarrhena asphodeloides Bge; 7. The dry roots of Acorus tatarinowii Schott; 8. The dry aboveground part of Pogostemon cablin (Blanco) Benth.; 9. The dry fruit of Forsythia suspensa (Thunb.) Vahl | Y |
| Jinzhen oral solution (Yan 2020) | 1. The horn of Saiga tatarica Linnaeus; 2. The dry bulb of Fritillaria ussuriensis Maxim.; 3. The dry roots of Rheum palmatum L.; 4. The dry mature fruit of Gardenia jasminoides Ellis; 5. Biotite Schist; 6. Gypsum Fibrosum (CaSO4·2H2O); 7. The artificial Bos taurus domesticus Gmelin; 8. The dry roots and rhizomes of Glycorrhiza uralensis Fisch., Glycorrhiza inflata Bat. or Glycorrhiza glabra L | Y |
| Reduning injection (Yan 2020） | 1. The dry aboveground part of Artemisia annua L; 2. The dry buds or with blooming flowers of Lonicera japonica Thunb.; 3. The dry mature fruit of Gardenia jasminoides Ellis | N |
| Xiyanping injection (Yan 2020) | The dried above-ground part of Andrographis paniculata (Burm.f.) Nees | N |
| Jinlianqingre effervescent tablets (Yan 2020) | 1. The horn of Saiga tatarica Linnaeus, 2. The dry roots of Bolbostemma paniculatum (Maxim.) Franquet； 3. The dry roots of Rheum palmatum L.; 4. The dry roots of Scutellaria baicalensis Georgi; 5. The dry gallstones of Bos taurus domesticus Gmelin; 6. Chalcanthitum; 7. Gypsum Fibrosum (CaSO4·2H2O); 8. The dry roots and rhizomes of Glycorrhiza uralensis Fisch., Glycorrhiza inflata Bat. or Glycorrhiza glabra L | N |
| Jinhua qinggan granule (Liu 2021) | 1. The dry caudex of Ephedra sinica Stapf, Ephedra intermedia Schrenk et C.A.Mey. or Ephedra equisetina Bge.； 2. The dry matured seeds of Prunus armeniaca L.var.ansu Maxim., Prunus sibirica L.or Prunus mandshurica (Maxim.) Koehne or Prunus armeniaca L.； 3. Gypsum Fibrosum (CaSO4·2H2O); 4. The dry roots and rhizomes of Glycorrhiza uralensis Fisch.； 5. The dry buds or with blooming flowers of Lonicera japonica Thunb.,； 6. The dry fruit of Forsythia suspensa (Thunb. ) Vahl; 7. The dry roots of Anemarrhena asphodeloides Bge.； 8. The dry roots of Scutellaria baicalensis Georgi; 9. The dry matured seeds of Arctium lappa L.； 10. The dry aboveground part of Mentha haplocalyx Briq.; 11. The dry aboveground part of Artemisia annua L.； 12. The dry roots of Fritillaria thunbergii Miq. | N |
| Lianhuaqingwen capsule (Liu 2021) | 1.The dry fruit of Forsythia suspensa (Thunb.) Vahl 255g；  2.The dry buds or with blooming flowers of Lonicera japonica Thunb.255g  3.The dry caudex of Ephedra sinica Stapf, Ephedra intermedia Schrenk et C.A.Mey. or Ephedra equisetina Bge. 85g  4.The dry matured seeds of Prunus armeniaca L.var.ansu Maxim., Prunus sibirica L.or Prunus mandshurica (Maxim.) Koehne or Prunus armeniaca L. 85g；  5.Gypsum Fibrosum (CaSO4·2H2O) 255g;  6.The dry roots of Isatis indigotica Fort.255g；  7.The dry roots of Dryopteris crassirhizoma Nakai 255g;  8. The dry aboveground part of Houttuynia cordata Thunb.255g;  9. The dry aboveground part of Pogostemon cablin (Blanco) Benth 85g;  10. The dry roots of Rheum palmatum L.51g;  11. The dry roots of Rhodiola crenulate (Hook. f. et Thoms.) H. Ohba 85g;  12. The fresh stem of Mentha haplocalyx Briq 7.5g;  13. The dry roots and rhizomes of Glycorrhiza uralensis Fisch., Glycorrhiza inflata Bat. or Glycorrhiza glabra L 85g. | Y |
| Shufeng Jiedu Capsule (Liu 2021) | 1.The dry roots of Polygonum cuspidatum Sieb. et Zucc.450g;  2.The dry fruit of Forsythia suspensa (Thunb.) Vahl 360g;  3.The dry roots of Isatis indigotica Fort. 360g;  4.The dry roots of Bupleurum chinense DC. or Bupleurum scorzonerifolium Willd.360g;  5.Patrinia scabiosaefolia 360g;  6.The dry aboveground part of Verbena officinalis L. 360g;  7.The fresh of dry roots of Phragmites communis Trin. 270g;  8.The dry roots and rhizomes of Glycorrhiza uralensis Fisch., Glycorrhiza inflata Bat. or Glycorrhiza glabra L.180g | Y |
| Xuebijing injection (Liu 2021) | 1.The dry flower of Carthamus tinctorius L.;  2. The dry roots of Paeonia lactiflora Pall. Or Paeonia veitchii Lynch;  3.The dry roots of Ligusticum chuanxiong Hort;  4. The dry roots of Salvia miltiorrhiza Bge.;  5.The dry roots of Angelica sinensis（Oliv.）Diels. | N |

**Notes:** CHM for Chinese herbal medicine; Y for yes; N for no.
