# Supplementary material for: Clinical Evidence on the Use of Chinese Herbal Medicine for Acute Infectious Diseases: An Overview of Systematic Reviews
Source: Front Pharmacol. 2022 Feb 25;13:752978. doi: 10.3389/fphar.2022.752978 (PMC8914111; doi:10.3389/fphar.2022.752978)
Supplement: Supplementary file 4 [file Table3.DOCX]

**Supplementary 3** Ingredients of the formulas

| **Study** | **Ingredients of the formulas** |
| --- | --- |
| Fan 2020 | NS |
| Pang 2020 | NS |
| Jin 2020 | NS |
| Luo 2020 | NS |
| Sun 2020 | NS |
| Zeng 2020 | NS |
| Wang 2020 | NS |
| Yang 2020 | NS |
| Ang 2020 | NS |
| Xiong 2020 | Gancao(Glycyrrhizae radix et rhizoma), Huangqin(Scutellariae radix), Banxia(Pinelliae rhizoma), Lianqiao(Forsythiae fructus), Kuxingren(Armeniacae Semen Amarum) |
| Liu 2020 | NS |
| Gao 2020 | NS |
| Liu 2020 | NS |
| Qi 2020 | NS |
| Wu 2020 | NS |
| Chen 2007 | NS |
| Liu 2004 | Herbal compounds No. I (Yiqi Yangyin), No. II (Bufei Jianpi), No. III (Yangyin Qingre); Herbal compounds Qingre Jiedu Shufeng Xuanfei or Yiqi Huayu Qingre Jiedu; Qiankunning (herbal compound of 14 herbs); Chuanhuning (herbal extracts) and Shenmai (herbal extracts); Herbal compounds ‘Guoyao’ No. 2, 3,or 4, depending on disease duration, one dosage daily; Qingkailing injection (herbal extracts), and Xuesaitong injection (ingredients of ginseng) |
| Liu 2012 | Compound herbs No. 1: Xiyangshen (Panax quinquenfolium L.) 6 g, Maidong (Opiopogan japonicus (Thunb.) Ker‐Gawl 12 g, Wuweizi (Schisandra chinensis (Turcz.) Baill 6 g, huangqi (Radix Astragalus membranacers) 18 g, Polygonatum odoratum (Mill.) Druce 12 g, Pollen 12 g, Baizhu (Atractylodes macrocephala Koidz.) 9 g, Fuling (Sclerotium Poriacocos) 12 g, Sangye (Morus alba L.) 12 g, danggui (Angelica sinensis) 9 g, Baishao (Paeonia lactiflora Pall.) 12 g,Chuanxiong (Ligusticum chuanxiong Hort.) 12 g, Heye (Nelumbo nucifera Gaertn.) 10 g, Liuyishan (Six-one powder)10 g;  Compound herbs No. 2: huangqi (Radix Astragalus membranacers) 30 g, Danshen (Radix Codonopsis pilosula) 15 g, baishu (Atractylodes macrocephala Koidz.) 15 g, Yufuling (Poria cocos (Schw.) 15 g, Caihu (Bupleurum chinense DC.) 9 g, Baishao (Paeonia lactiflora Pall.) 12 g, Danggui (Angelica sinensis) 9 g, Muxiang (Aucklandia lappa Decne) 12 g, Sharen (Amomum villosum Lour) 6 g, Chenpi (Citrus reticulate Blanco) 12 g, Qingbanxia (Pinellia ternate (Thunb Breit) 9 g, Huoxiang (Agastache rugosa (Fisch. Et Mey.) O.Ktze ) 10 g, Jiaosanxian (Hordei fructus germinatus,Crataegi fructus,Massa medicata fermentata)10 g;  Compound herbs No. 3: Xiyangshen (Panax quinquenfolium L.) 3 g, Shashen (Glehniae radix)15 g, Maidong (Ophiopogon japonicus (Thunb.) Ker‐Gawl.) 12 g, Caihu (Bupleurum chinense DC.) 9 g, Huangqi (Radix Astragalus membranacers) 12 g, Sangye or white mulberry leaf (Morus alba L.) 15 g, Sangpi or white myberry bark (Morus alba L.) 15 g, Digupi (Lyrmeleon chinense Mill.) 12 g, Qinghao (Attenmisa annua L.) 15 g, Lugen (Phragmites communis Trin.) 15 g, Maogen (Imperata cylindrica Beauv. Var. major (Nees) C.E.Hubb.) 15 g, Danggui (Angelica sinensis (Oliv. Diels)) 9 g, Baishao (Paeonia lactiflora Pall.) 12 g, Qingbanxia (Pinellia ternate (Thumb Breit.) 9 g, maiya (Hordeum vulgare L.) 15 g, guya (Oryza sativa L.) 15 g  National drug No. 2: shigao(Gypsum fibrosum), Huangqin (Scutellaria baicalensis), Zicao (Lithospermum erythrorhizon Sieb. Er Zucc);  National drug No. 3: Dihuang(Rehmanniae radix), Xuanshen(Scrophulariae radix),Jinyinhua (Loinicera japonica Thunb.)  National drug No. 4: Taizishen (Pseudostellaria heterophylla (Miq.) Pax er Pax er Hoffm), Shashen (Glehniae radix), dangsheng (Loinicera japonica Thunb.)  Kangfeidian No. 1, 2, 3: No. 1 of Feidian: shigao (Gypsum fibrosum)45 g, Caihu (Bupleuri radix)15 g, Zhimu (Anemarrhenae rhizoma)10 g, Beimu (Fritillariae thunbergii bulbus)10 g, Huangqin (Scutellariae radix)15 g, Qinghao (Artemisiae annuae herba)15 g, Danpi (Moutan cortex)10 g, Chishao (Paeoniae radix rubra)12 g, Lianqiao (Forsythiae fructus)15g, Shanyurou (Corni fructus)30 g, Cangzhu (Atractylodis rhizoma)15 g, Huoxiang (Pogostemonis herba)10 g, Yiyiren （Coicis semen）15 g, xingren （Armeniacae semen amarum）10 g; No. 2 of Feidian: Huangqin (Scutellariae radix)15 g, Qinghao （Artemisiae annuae herba）15 g, Gualou （Trichosanthis fructus）30 g, Dangshen （Codonopsis radix）15 g, Xuanfuhua （Inulae flos）10 g, Yujin （Curcumae radix）10 g, Shichangpu (Acori tatarinowii rhizoma)10 g, Bixie(Diocscorea hypoglauca Palibin)12 g, Cansha (Silkworm shit)15 g, Cangzhu (Atractylodis Rhizoma)15 g, Baizhu (Atractylodis Macrocephalae Rhizoma)15 g, Zhuling (Polyporus)15 g, Fuling (Poria)15 g, Yiyiren (Coicis Semen)15 g, Kuxingren (Armeniacae Semen Amarum )10 g, Cheqianzi (Plantaginis Semen)10 g, Shanyurou (Corni fructus)30 g; No. 3 of Feidian: Xiyangshen (Panacis Quinquefolii Radix)30 g, huangqi (Astragali Radix)30 g, Shanyurou (Corni fructus)30 g, Maidong (Ophiopogonis radix)15 g, Zhimu (Anemarrhenae Rhizoma)10 g, Beimu (Fritillariae Thunbergii Bulbus)10 g, Baijiangcan(Bombyx Batryticatus) 30 g, Lianqiao (Forsythiae Fructus)15 g, Danshen (Salviae Miltiorrhizae Radix Et Rhizoma)15 g, Picao 12 g, Cansha (Silkworm shit)15 g, Yiyiren (Coicis Semen)15 g, Zhuling (Polyporus)15 g, Fuling (Poria)15 g, Gualou (Trichosanthis fructus)30 g, Ziwan (Asteris Radix Et Rhizoma)15 g;  Compound Herbal formulas: When the participants had a fever, the following formula was used:mahuang (Ephedra sinica Stapf.) 5 g, Xingren (Prunus armeniaca L.) 12 g, Shigao (Gypsum fibrosum)45 g, Zhimu (Anemarrhena asphodeloides Bge.) 10 g, Jinyinhua (Loinicera japonica Thunb.) 15 g, Lianqiao (Forsythia suspense (Thumb Vahl) 12 g, zhizi (Gardenia jasminoides Ellis) 12 g, Huangqin (Scutellaria baicalensis Georgi) 12 g, Zisuye(Perillae folium)10 g, Yinchen （Artemisiae scopariae herba）15 g, Gegen (Pueraria edulis Pamp.)15 g, Taizishen (Pseudostellaria heterophylla (Miq.)) 15 g  When the participants had a cough, the following formula was used: Xiyangshen (Panax quinquenfolium L.) 15 g, Maidong (Ophiopogon japonicus (Thunb.) Ker‐Gawl.) 10 g, Wuweizi (Schisandra chinensis (Turcz.) Baill.) 10 g, Shanyurou (Cornus officinalis Sieb.et Zucc.) 12 g, Tinglizi (Descurainia Sophia (L.) Webb ex Prantl) 15 g,Ziwan (Aster tatarisuc L.f.) 15 g, Cebaiye (Eriobotrya japonica (Thumb.) Lindl.) 12 g, Dilong (Allobophora caliginosa (Savigny) trapezoids (Ant.Duges)) 12 g, Danshen (Salviae Miltiorrhizae Radix Et Rhizoma)12 g, Chishao (Paeoniae radix rubra)12 g, Jinyinhua (Loinicera japonica Thunb.) 8 g, Huanqin (Scutellaria baicalensis) 10 g, Gualoupi (Trichosanthes kirilowii Maxim) 15 g, Gegen (Pueraria edulis Pamp.)  When the participants were in the convalescence stage, the following formula was used: Taizisheng (Pseudostellaria heterophylla (Miq.)) 15 g, Maidong (Ophiopogon japonicus (Thunb.) Ker‐Gawl.) 15 g, Shashen (Glehniae radix)15 g, Baizhu (Atractylodes macrocephala Koidz.) 15 g, Cebaiye(Platycladi cacumen)15 g, Sharen (Amomum villosum Lour.) 6 g,Jiaosanxian (Hordei fructus germinatus,Crataegi fructus,Massa medicata fermentata)30 g, Shenghuangqi (Radix Astragalus membranacers) 15 g, Gegen (Pueraria edulis Pamp.) 15 g, Danshen（Salviae miltiorrhizae radix et rhizoma）15 g, Chenpi(Citrus reticulate Blanco) 6 g, Huangjing (Polygonatum sibiricum Red.) 15 g  6 Chinese traditional patient medicines: Chuanghuning injection, Shengmai injection, Hufei Qingsha drink, Jiedu Zhitong capsule, Zhuyin Sanjie capsule, Qingshaling spray;  Compound Chinese herbs (x): 1. Yi Qi Yang Ying recipe: Xiyangshen (Panax quinquenfolium L.), Maidong (Ophiopogon japonicus (Thumb Ker‐Gawl.), Wuweizi (Schisandra chinensis (Turcz.), huangqi (Radix Astragalus membranacers), Yuzhun(Polygonati odorati rhizoma), Tianhuafen(Trichosanthis radix) et al  2. Bu Fei Jian Pi recipe: huangqi (Radix Astragalus membranacers), Danshen (Radix Codonopsis pilosula), Baizhu (Atractylodes macrocephala Koidz.), Yufuling (Poria cocos (Schw.), Caihu (Bupleurum chinense DC.), Baishao (Paeonia lactiflora Pall.), Danggui (Angelica sinensis) et al  3. Yang Yin Qing Re recipe: Xiyangshen (Panax quinquenfolium L.) , Shashen（Amomi fructus）, Maidong (Opiopogan japonicus (Thunb.) Ker‐Gawl, Caihu (Bupleurum chinense DC.), Huangqin (Scutellaria baicalensis Georgi), Sangye(Mori folium), Digupi (Lyrmeleon chinense Mill.), Qinghao (Attenmisa annua L.).  Compound Chinese herbs (z): 1. Yi Qi Yang Ying recipe: Taizishen (Pseudosterllari heterophylla (Miq.) Pax er Pax er Hoffm) 30 g, Maidong (Ophiopogon japonicus (Thumb.) Ker‐Gawl.) 12 g, Wuweizi (Schisandra chinensis (Turcz.) 6 g, huangqi (Radix Astragalus membranacers) 15 g, Huafen(Trichosanthis radix)12 g, Fuling (Sclerotium Poriacocos) 12 g, Chuangxiong (Ligusticum chuanxiong Hort.) 12 g, Dangui (Angelica sinenesis) 9 g, Baishao (Paeonia lactiflora Pall) 9 g, Baizhu (Atractylodes macrocephala KoidZ) 15 g, Gancao (Glycyrrhizae radix et rhizoma) 9 g, Caihu (Bupleurum chinense DC.) 12g, Liuyishan (Six-one powder)10g  2. Bu Fei Jian Pi recipe: huangqi (Radix Astragalus membranacers) 20 g, Danshen (Radix Codonopsis pilosula) 15 g, Chaobaishu (Atractylodes macrocephala Koidz.) 15 g, Yufuling (Poria cocos(Schw.) 15 g, Caihu (Bupleurum chinense DC.) 9 g, Danggui (Angelica sinensis) 9 g, Baishao (Paeonia lactiflora Pall.) 12 g, Chuanxiong (Ligusticum chuanxiong Hort.) 12 g, Muxiang (Aucklandia lappa Decne) 12 g, Chenpi (Citrus reticulate Blanco) 12 g, Huoxiang (Agastache rugosa (Fisch. Et Mey) 10 g, Jiaosanxian(Hordei fructus germinatus,Crataegi fructus,Massa medicata fermentata) 10 g, Sharen (Amomum villosum Lour.) 6 g  3. Yang Yin Qing Re recipe: Taizishen (Pseudostellaria heterophylla (Miq.) Pax er Hoffm) 15 g, Shashen(Glehniae radix)15 g, Maidong (Opiopogan japonicus (Thunb.) Ker‐Gawl ) 12 g, Caihu (Bupleurum chinense DC.) 9 g, Sangye (Morus alba L.) 15 g, Sangpi (Morus alba L.) 15 g, Huangqin (Scutellaria baicalensis Georgi) Digupi (Lyrmeleon chinense Mill.) 12 g, Qinghao (Attenmisa annua L.) 15 g, Lugen (Phragmites communis Trin) 15 g, Lugen (Phragmites communis Trin) 15 g, Qingbanxia (Pinellia ternate (Thumb.) Breit) 9 g, Baishao (Paeonia lactiflora Pall.) 12 g, Danggui (Angelica sinensis) 9 g, Huangqi (Radix Astragalus membranacers) 12 g, Liuyishan (Six-one powder)10 g, Mudanpi (Salviae miltiorrhizae radix et rhizoma)12 g  Compound Chinese herbs (j): 1. Yingqing heji (for normal SARS participants): Jinyinhua (Loinicera japonica Thumb) 20 g, Daqingye (Isatidis folium)20 g, Guanzhong (Dryopteridis crassirhizomatis rhizoma)15 g, Gegen (Pueraria edulis Pamp) 15 g, Zisuye(Perillae folium)12 g, Jiegeng (Platycodonis radix)15 g, Huoxiang (Agastache rugosa (Fisch.Et Mey.) 15 g, Gancao (Glycyrrhizae radix et rhizoma)30 g  2. Compound Yuxing heji (for severe and acute sever SARS patients): Yuxingcao(Houttuyniae herba) 45 g, Banlangen (Isatidis radix)45 g, Huangqin (Scutellaria baicalensis) 15 g, Kuxingren (Armeniacae semen amarum)15 g, Caihu (Bupleurum chinense DC.) 15 g, Qinghao (Attenmisa annua L.) 15 g, Xianhecao (Agrimoniae herba)20 g, Zhuru (Bambusae caulis in taenias)15 g, Shigao (Gypsum fibrosum)30 g, Zhimu (Anemarrhena asphodeloides Bge.) 20 g, Taizishen (Pseudostellaria heterophylla (Miq) Pax er Hoffm) 20 g, Gancao (Glycyrrhizae radix et rhizoma) 30 g  3. Ganqi heji (for SARS patients of recovery stage): Huangqi (Angelica sinensis) 45 g, Gancao (Glycyrrhizae radix et rhizoma) 30 g, Taoren(Persicae semen) 30 g, Biejia (Trionycis carapax)30 g  4. Kangfeidiang No. 1 granule: Guangzhong (Dryopteridis crassirhizomatis rhizoma)20 g, Chaihu (Bupleurum chinense DC.) 10 g, Wuwezi (Schisandra chinensis (Turcz.) Baill) 6 g  5. Kangfeidian No. 2 granule: Jinyinhua (Loinicera japonica Thunb.)10 g, Guangzhong (Dryopteridis crassirhizomatis rhizoma) 10 g, Daqingye (Isatidis folium)10 g  Compound Chinese herbs (y): 1. Shigao (Gypsum fibrosum)30 to 50 g, Zhimu (Anemarrhena asphodeloides Bge.) 10 g, Jinyinhua (Loincera japonica Thunb.) 30 g, Rendongteng (Lonice raejaponicae caulis)30 g, Lianqiao (Forsythia suspense (Thumb.) Vahl) 10 g, Qianghuo (Notopterygii rhizoma et radix)10 g, Bohe (Menthae haplocalycis herba)10 g, Gancao (Glycyrrhizae radix et rhizoma) 6 g, Lingyangjiao (Saigae tataricae cornu)0.3 g etc.  2. Huangqi (Scutellaria baicalensis Georgi) 15 to 30 g, Shigao (Gypsum fibrosum)30 g, Lingyangjiao (Saigae tataricae cornu)0.6 g, Tianzhuhuang (Bambusae concretio silicea)10 g, Danshen (Salviae miltiorrhizae radix et rhizoma)15 to 30 g,Sanqi (Notoginseng radix et rhizoma)3 g  3. Taizishen (Pseudostellaria heterphylla (Miq) Pax er Hoffm) 15 g, Huangqi (Scutellaria baicalensis Georgi) 15 to 30 g, Cangzhu (Atractylodis rhizoma)10 g, Baizhu (Atractylodis Macrocephalae Rhizoma)10 g, Baibiandou (Lablab semen album)30 g,Yiyiren (Coicis Semen)30 g, Gualoupi (Trichosanthes kirilowii Maxim) 10 g, Sigualuo (Luffae fructus retinervus)10 g, Danshen (Salviae miltiorrhizae radix et rhizoma)30 g, Cebaiye(Platycladi cacumen)10 g  Compound Chinese herbs (ls): When treatment was in progress, the following recipe was used: Loulu (Rhapontici radix)15 g,Lianqiao (Forsythia suspense (Thumb.) Vahl) 12 g, Jinyinhua (Loinicera japonica Thumb.) 15 g, Huangqin (Scutellaria baicalensis) 10 g, Qinghao (Attenmisa annua L.) 15 g, Shigao (Gypsum fibrosum)30 g, Gualoupi (Trichosanthes kirilowii Maxim) 15 g, Beimu (Fritillariae Thunbergii Bulbus)12 g, Zhizi (Gardenia jasminoides Ellis)10 g, Cheqianzi (Plantaginis Semen)10 g (package), Chishao (Paeoniae radix rubra)12 g  2. When patients were at recovery stage, the following recipe was used: Bailing capsule 15 g, Taizishen (Pseudostellaria hetrophylla (Miq.)) Paxer Hoffm) 20 g, Xuanshen (Scrophulariae radix)12 g, Chishao (Paeoniae radix rubra)12 g, Loulu (Rhapontici radix)15 g, Lianqiao (Forsythia suspense (Thumb.) Vahl) 12 g, Sanqi (Notoginseng radix et rhizoma)3 g, Jiaosanxian (tus baking Fructus hordei germinatus et crataegi massa fermentataa medicinalis) 10 g, Zhike (Aurantii fructus)10 g, Yiyiren (Coicis Semen)30 g |
| Zhang 2004 | NS |
| Hao 2005 | NS |
| Hao 2005 | NS |
| Liu 2005 | NS |
| Zhao 2004 | NS |
| Zhao 2014 | NS |
| Li 2016 | NS |
| Jin 2018 | NS |
| Yan 2017 | NS |
| Yue 2017 | NS |
| Guo 2010 | NS |
| Wang 2017 | NS |
| Han 2016 | NS |
| Wu 2015 | NS |
| Zhang 2016 | NS |
| Zhao 2014 | NS |
| Lu 2013 | NS |
| Liu 2016 | NS |
| Zhang 2014 | NS |
| Zhang 2014 | NS |
| Xiong 2013 | NS |
| Wang 2013 | NS |
| Ding 2013 | NS |
| Yu 2020 | NS |
| Yang 2020 | NS |
| Yan 2020 | Kangbingdu oral solution: Banlangen(Isatis root), Shigao(Gypsum fibrosum), Lugen(Phragmitis rhizoma), Dihuang(Rehmanniae radix), Yujin(Curcumae radix), Zhimu(Anemarrhenae Rhizoma),Shichangpu(Acori tatarinowii rhizoma), Guanghuoxiang(Pogostemonis herba), Lianqiao(Forsythiae Fructus);  Jinzhen oral solution: Lingyangjiao (Saigae tataricae cornu), Beimu(Fritillariae Thunbergii Bulbus), Dahuang(Rhei radix et rhizoma), Huangqin(Scutellariae radix),Shigao(Gypsum fibrosum),Niuhuang(Bovis calculus),Gancao( liquorice);  Reduning injection: Qinghao(Artemisiae annuae herba), Jinyinhua(Lonicerae japonicae flos) and Zhizi(Gardeniae fructus);  Xiyanping injection: Total ester sulfonate of andrographolide;  Jinlianqingre effervescent tablets: Lingyangjiao (Saigae tataricae cornu), Beimu(Fritillariae Thunbergii Bulbus), Dahuang(Rhei radix et rhizoma), Huangqin(Scutellariae radix)，Niuhuang(Bovis calculus), Danfan(Bluestone), Shigao(Gypsum fibrosum), Gancao( Glycyrrhizae radix et rhizoma) |
| Xiong 2019 | NS |
| He 2020 | NS |
| Zhou 2022 | NS |
| Liu 2021 | Jinhua qinggan granule: Maxingshigantang，Jinyinhua(Lonicerae japonicae flos)，Lianqiao(Forsythiae Fructus), Zhimu(Anemarrhenae Rhizoma), Huangqin(Scutellariae Radix), Niubangzi(Arctium lappa), Bohe(Mentha haplocalyx), Qinghao (Artemisiae annuae herba), Beimu(Fritillaria thunbergii )  Lianhuaqingwen capsule: Maxingshigantang，Jinyinhua(Lonicerae japonicae flos)，Lianqiao(Forsythiae Fructus), Banlangen(Isatis root), Mianmaguanzhong, Yuxingcao(Houttuynia Herba), Guanghuoxiang(Pogostemonis herba), Dahuang (Rhei radix et rhizoma), Hongjingtian(Rhodiola crenulata), Bohenao(Mentha haplocalyx)  Shufeng Jiedu Capsule: Huzhang(Polygoni cuspidati rhizoma et RADIX), Lianqiao(Forsythiae Fructus), Banlangen(Isatis root), Chaihu (Bupleurum chinense DC.), Baijiangcan(Bombyx Batryticatus), Mabiancao(Verbena officinalis L), Lugen(Phragmites communis Trin), Gancao(Glycyrrhizae radix et rhizoma)  Xuebijing injection: Honghua(Carthamus tinctorius L), Chishao(Paeonia lactiflora Pall.), Chuanxiong(Ligusticum chuanxiong Hort.), Dancan, Danggui (Angelica sinensis) |
| Zhou 2021 | NS |
| Ouyang 2021 | NS |
| Yu 2020 | NS |

NS: Not stated
