# Supplementary material for: Clinical Evidence on the Use of Chinese Herbal Medicine for Acute Infectious Diseases: An Overview of Systematic Reviews
Source: Front Pharmacol. 2022 Feb 25;13:752978. doi: 10.3389/fphar.2022.752978 (PMC8914111; doi:10.3389/fphar.2022.752978)
Supplement: Supplementary file 5 [file Table2.doc]

**Supplementary 2** excluded references

[1]Zeng HY,Chen YT,He YY,Gu YQ,Chen XF.Identification of components in vitro and plasma of JHQG (Jinhua Qinggan Granules) and preliminary pharmacokinetics analysis[J/OL].Acta Pharmaceutica Sinica:1-15[2021-11-22].http://kns.cnki.net/kcms/detail/11.2163.R.20211118.2254.007.html.

[2]Li YH,Ren XF,Han L,Di YH,Zhao WD,Chang YM,Zhang PQ,Kang BB.Meta-analysis of the diagnostiefficacy of SARS-CoV-2 specific IgM and IgG antibodies for COVID-19[J].Clinical Research and Practice,2021,6(25):10-15.[3]Zeng HY,He YY,Tang QL,Li K,Gu YQ,Chen XF.Research progress on chemicalcomposition and clinical efficacy of Lianhua Qingwen (LHQW) capsule, a traditional Chinese medicine (TCM) used to treat COVID-19[J].Journal of Pharmaceutical Practice,2021,39(04):291-294+361.

[4]Gao A,Lu SW,Chen L,Gan JH,Sun W.Safety of corticosteroids in management of severe coronavirudisease 2019 cases: a meta-analysis[J].Chinese Journal of Infection and Chemotherapy,2021,21(04):411-417.[5]Xu T,Yang L,Gao YF,Zhang JP.SARS-CoV-2 Nucleic Acid Positive Rate among Close Contacts of the Patients Infected with Novel Coronavirus in China:A Meta-Analysis[J].Journal of Modern Laboratory Medicine,2021,36(04):122-128.

[6]Liu Y. CLINICAL MANIFESTATIONS AND OUTCOMES OFPREGNANT WOMEN WITH COVID-19: A SYSTEMATICREVIEW AND META-ANALYSIS[D].Chongqing Medical University,2021.

[7]Wu XY,Wu XP,Bai X.Systematic Review of Efficacy and Safety of Lopinavir /Ritonavir in Patients with COVID －19[J].Evaluation and Analysis of Drug-Use in Hospitals of China,2021,21(04):473-477.

[8]Meng JY,Li ZM,Huang MM,Li JX.Systematic Evaluation on the External Therapy of Traditional Chinese Medicine in the Treatment of Ｒheumatoid Arthritis[J].Guangming Journal of Chinese Medicine,2021,36(05):691-695.

[9]Zhang Y,Sun Y,Duan W,Ma CN,Wu SS,Zhang DT,Wang QY.Meta analysis of the rate of nucleic acid recovery after discharge from New Coronavirus pneumonia in Medical University Of Chongqing[J].International Journal of Virology,2021,28(01):6-10.[10]Liu LL,Duan F,Du QT,Cui CH.A review and Meta-analysis of clinical efficacy and safety of the integrative medicine on COVID-19[J].Clinical Journal of Chinese Medicine,2021,13(05):24-30.

[11]Fu CY,Li JB,Liu GX.Incidence and risk factors of acute kidney injury in patients with New Coronavirus pneumonia: a Meta analysis[J].Chongqing Medicine,2021,50(09):1562-1567.

[12]Xu Y,Yan YB.Meta analysis of Pudilan Xiaoyan oral liquid in the treatment of herpetic angina in children[J].China's Naturopathy,2020,28(22):52-55.

[13]Zhang SQ,Jia M,Cai X.Meta-analysis of Systemic Lupus Erythematosus Treated with Integrated Chinese and Western Medicine ZHANG Shao-qin,JIA Min,CAI Xin[J].Rheumatism and Arthritis,2020,9(11):28-33+38.

[14]Wu ZY,Du X,Cheng K,Cheng YC,Wang ZY,Zhai SD,Liu W,Xie XH.Systematic Ｒeview on Efficacy and Safety of Ｒibavirin in the Treatment of Severe Acute Respiratory Syndrome and Middle East Ｒespiratory Syndrome[J].Evaluation and Analysis of Drug-Use in Hospitals of China,2020,20(10):1236-1239.

[15]Shun KY,Wang HJ,Zhao C.Susceptibility to severe acute respiratory syndrome coronavirus type 2 in patients with hypertension and the use of angiotensin converting enzyme inhibitors[J].Journal of Microbes and Infections,2020,15(05):316-321.

[16]Shao Y,Shi HJ,Tang W.Meta analysis of clinical efficacy of TCM Massage in the treatment of acute mastitis[J].Lishizhen Medicine and Materia Medica Research,2020,31(08):2046-2048.

[17]Lai F,Ren Y,Lai ZC,Zeng RF,Li J.Acupuncture at "Zusanli"(ST36) for Acute Lung Injury in ExperimentalSepsis: A Meta-Analysis[J].Guiding Journal of Traditional Chinese Medicine and Pharmacy,2020,26(11):124-130.

[18]Cheng XL. Meta-analysis of the Study on the Intervention of Traditional Chinese Medicine in the Breast Cancer Precancerous Lesions and the Expression of EMT Markers in Benign and Malignant Breast Tissues.Shandong University of traditional Chinese Medicine,2020.

[19]Li XL,Liu WR,He WQ,Xie Y,Li JS.Meta analysis of TCM Syndrome Distribution in 2139 cases of New Coronavirus pneumonia[J].Chinese Critical Care Medicine,2020,32(06):664-670.

[20]Gao CY,Song CM,Fu YL,Zhang J.Curative Effect on Treating COVID-19 by Integrated Medicine: A Systematic Review[J].Journal of Shanxi University of Chinese Medicine,2021,44(01):1-9.

[21]Lou SP, Liu PY, Chen H. Risk factors of severe and critical coronavirus disease 2019: a meta-analysis[J]. Journal of Third Military Medical University,2020,42(14):1469-1475.

[22]Liu YR, Tang ZS, Song ZX. Feasibility Analysis of siji Kangbingdu Mixture for Treatment of Children COVID-19[J]. Modern Chinese Medicine,2020,22(04):492-503.

[23]Wu YY, Li HY, Xu XB.Clinical features and outcome of New Coronavirus pneumonia: a meta-analysis[J/OL]. Chinese Journal of Hepatology,2020(03):240-241-242-243-244-245-246[2021-11-22].

[24]Li X, Feng AZ, Ma W. Traditional Chinese Medicine Syndromes of the Novel Coronavious Pneumonica: A Systemic Review and Meta-analysis[J]. World Chinese Medicine,2020,15(03):305-309+314.

[25Ge YL, Wang R, Cang HL.Epidemiology, clinical characteristics and discharge outcomes of children with COVID-19 based on eight case series studies and 10 case reports: A systematic review[J]. Chinese Journal of Evidence-Based Pediatrics,2020,15(01):25-31.

[26]Liu Y, Liu L, Cao MM. Literature analysis of Chinese patent medicine treatment in the period of Clinical Management of Corona Virus Disease 2019(trial 6th edition)[J]. Clinical Medication Journal,2020,18(02):62-66.

[27]Ma GH, Tian LM, Wang Y. Systematic evaluation and meta-analysis of integrated traditional Chinese and Western medicine in the treatment of MLR-IYB [J]. Chinese Journal of Antituberculosis,2020,42(02):95-100.

[28]Williams Phoebe C M,Berkley James A. Guidelines for the treatment of dysentery (shigellosis): a systematic review of the evidence.[J]. Paediatrics and international child health,2018,38(sup1):

[29]Cao WH.Lianhua Qingwen capsule and its basic research and clinical application meta analysis[J]. Biped and Health,2018,27(17):171-172.

[30]Yi F. Meta analysis of four traditional Chinese medicine injections in the treatment of children's hand, foot and mouth disease[D].Yichun University,2018.

[31]Ruohan Wu,Shigang Liu,Jin Sun,Lily Lai,Jianping Liu. Chinese herbal medicine for hand-foot-and-mouth disease in children: An overview of systematic reviews[J]. Journal of Traditional Chinese Medical Sciences,2018,5(2):

[32]Yue JB, Xiong L, Wang CQ.Systematic evaluation of the effect of Astragalus Membranaceus Injection on reducing toxicity and increasing efficiency of conventional antituberculous chemotherapy drugs[J]. Shandong Medical Journal,2017,57(24):74-77.

[33]Yang SY. Meta analysis of integrated traditional Chinese and Western medicine in the treatment ofprimary pulmonary tuberculosis[D].Zhejiang Chinese Medicine University,2017.

[34]Yue JB, Xiong L, Wang CQ. Meta － analysis on Synergy and Attenuation of Astragalus Membranaceus Ｒelated Oral Preparations of Traditional

Chinese Medicine in the Adjuvant Therapy for Pulmonary Tuberculosis [J]. Practical Journal of Cardiac Cerebral Pneumal and Vascular Disease,2017,25(04):1-7.

[35]Zhang YW. Research on the prevention and treatment of viral diseases in TCMbased on Meta analysis[D].Shandong University of traditional Chinese Medicine,2016.

[36]Pan JW. A Systematic Review about Traditional Chinese Medicine Treatment of Viral Pneumonia[D].Guangzhou University of traditional Chinese Medicine,2016.

[37]Li JK, Liu Y, Wang YL.Meta analysis of traditional Chinese medicine or integrated traditional Chinese and Western medicine in the treatment of infectious diseases of fever with respiratory syndrome[J]. Medical Equipment,2015,28(17):101-103.

[38]Sun H. Literature Research and Systematic Reviews on Property and Clinical Application of Honeysuckle[D].Hunan University of traditional Chinese Medicine,2015.

[39]Zhang RX, Liu WW. Meta-analysis of efficacy of Oseltamivir and substitution therapy for anti- H1N1 infection [J]. China Medical Herald,2014,11(31):52-55+60.

[40]Cao HJ,Liu ZL,Peter Steinmann,Mu YJ,Luo H,Liu JP. Chinese herbal medicines for treatment of hand, foot and mouth disease: A systematic review of randomized clinical trials[J]. European Journal of Integrative Medicine,2012,4(1)
